# Single-cell, single-nucleus and xenium-based spatial transcriptomics analyses reveal inflammatory activation and altered cell interactions in the hippocampus in mice with temporal lobe epilepsy

**DOI:** 10.1186/s40364-024-00636-3

**Published:** 2024-09-13

**Authors:** Quanlei Liu, Chunhao Shen, Yang Dai, Ting Tang, Changkai Hou, Hongyi Yang, Yihe Wang, Jinkun Xu, Yongchang Lu, Yunming Wang, Yongzhi Shan, Penghu Wei, Guoguang Zhao

**Affiliations:** 1 Department of Neurosurgery, Xuanwu Hospital Capital Medical University, 54 Changchun Street, Xicheng District, Beijing, 100053 China; 2 Brain Research Innovation and Translation Laboratory, Xuanwu Hospital Capital Medical University, 54 Changchun Street, Xicheng District, Beijing, 100053 China; 3 Clinical Research Centerfor, Epilepsy Capital Medical University, 54 Changchun Street, Xicheng District, Beijing, 100053 China

**Keywords:** Temporal lobe epilepsy, Single-cell RNA sequencing, Single-nucleus RNA sequencing, Xenium-based spatial transcriptomics

## Abstract

**Background:**

Temporal lobe epilepsy (TLE) is among the most common types of epilepsy and often leads to cognitive, emotional, and psychiatric issues due to the frequent seizures. A notable pathological change related to TLE is hippocampal sclerosis (HS), which is characterized by neuronal loss, gliosis, and an increased neuron fibre density. The mechanisms underlying TLE-HS development remain unclear, but the reactive transcriptomic changes in glial cells and neurons of the hippocampus post-epileptogenesis may provide insights.

**Methods:**

To induce TLE, 200 nl of kainic acid (KA) was stereotactically injected into the hippocampal CA1 region of mice, followed by a 7-day postinjection period. Single-cell RNA sequencing (ScRNA-seq), single-nucleus RNA sequencing (SnRNA-seq), and Xenium-based spatial transcriptomics analyses were employed to evaluate the changes in mRNA expression in glial cells and neurons.

**Results:**

From the ScRNA-seq and SnRNA-seq data, 31,390 glial cells and 48,221 neuronal nuclei were identified. Analysis of the differentially expressed genes (DEGs) revealed significant transcriptomic alterations in the hippocampal cells of mice with TLE, affecting hundreds to thousands of mRNAs and their signalling pathways. Enrichment analysis indicated notable activation of stress and inflammatory pathways in the TLE hippocampus, while pathways related to axonal development and neural support were suppressed. Xenium analysis demonstrated the expression of all 247 genes across mouse brain sections, revealing the spatial distributions of their expression in 27 cell types. Integrated analysis of the DEGs identified via the three sequencing techniques revealed that *Spp1*, *Trem2*, and *Cd68* were upregulated in all glial cell types and in the Xenium data; *Penk*, *Sorcs3*, and *Plekha2* were upregulated in all neuronal cell types and in the Xenium data; and *Tle4* and *Sipa1l3* were downregulated in all glial cell types and in the Xenium data.

**Conclusion:**

In this study, a high-resolution single-cell transcriptomic atlas of the hippocampus in mice with TLE was established, revealing potential intrinsic mechanisms driving TLE-associated inflammatory activation and altered cell interactions. These findings provide valuable insights for further exploration of HS development and epileptogenesis.

**Supplementary Information:**

The online version contains supplementary material available at 10.1186/s40364-024-00636-3.

## Introduction

**Fig. 1:**
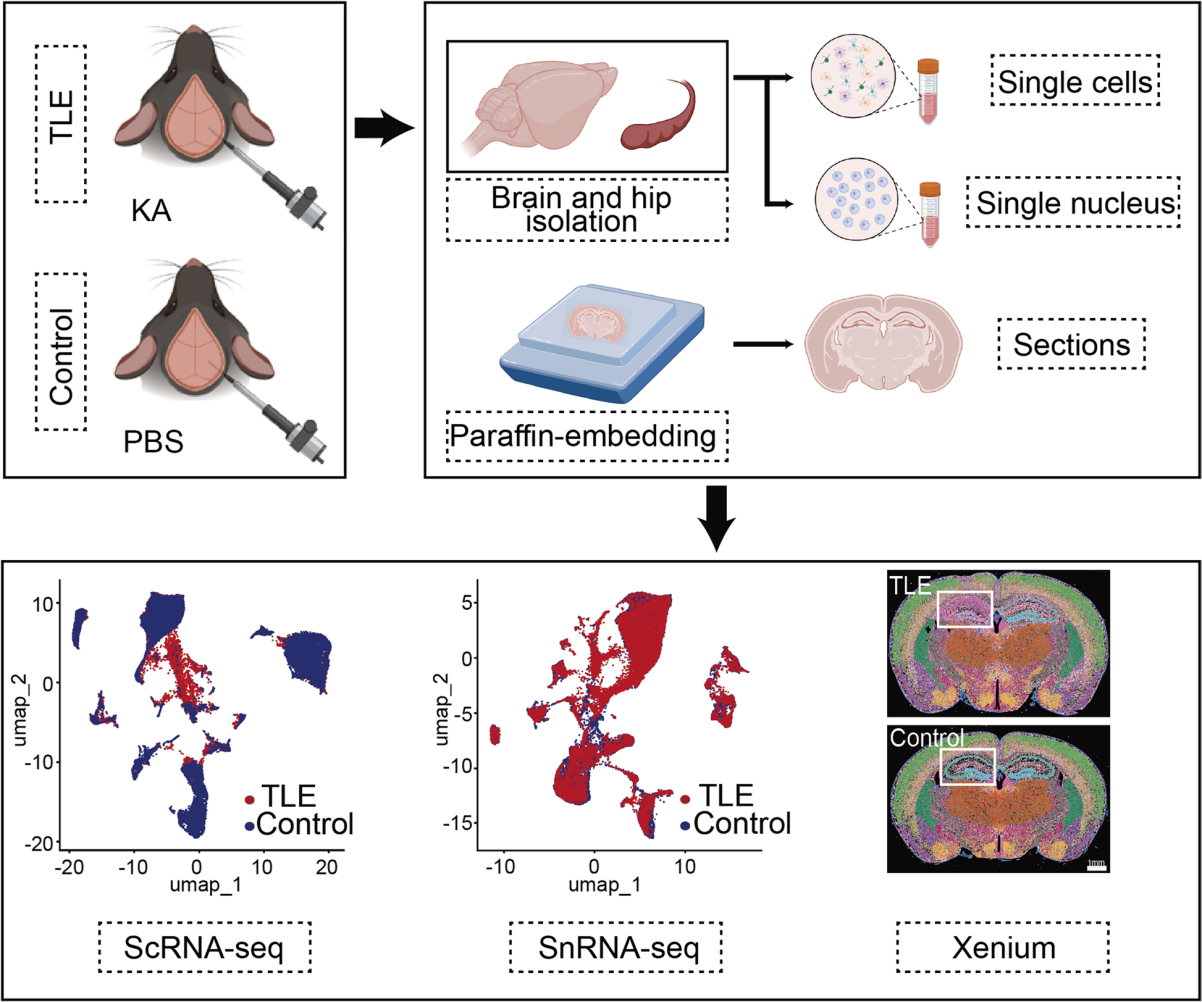
Flow chart of this study (Created with BioRender.com)

Temporal lobe epilepsy (TLE) accounts for 40% to 60% of all focal epilepsy cases and is thus one of the most clinically prevalent forms of epilepsy. Patients with TLE exhibit complex clinical manifestations, including recurrent motor seizures often accompanied by transient loss of consciousness. The frequent seizures can also lead to cognitive, emotional, and psychiatric issues [1, 2]. The precise mechanisms underlying TLE remain unclear, but factors such as genetics, trauma, inflammation, and neurotransmitter imbalances are believed to contribute to the disruption of excitatory–inhibitory balance [3–5]. The most notable and common pathological changes related to TLE are observed in the hippocampus and are typically characterized by neuronal loss, glial proliferation, and an increased density of nerve fibres, a constellation collectively termed hippocampal sclerosis (HS) [6, 7]. During the progression of HS, there is a continuous loss of various hippocampal neurons, particularly interneurons, which directly leads to localized hyperexcitability in the hippocampus [8]. Additionally, aberrant neurogenesis or mossy fiber sprouting further exacerbates seizure activity by abnormally reconstructing local neural circuits [9, 10]. In recent years, numerous studies have suggested that local inflammatory responses can lead to hippocampal neuron damage and seizures. In particular, activated microglia and astrocytes may play a critical role in this process [11, 12].To investigate the endogenous mechanisms driving TLE-HS and hippocampal hyperexcitability, we aimed to construct an ultra-high-resolution transcriptomic atlas of the hippocampus of mice with TLE using single-cell, single-nucleus, and Xenium-based spatial transcriptomics techniques (Fig. 1). This atlas will allow the analysis of changes in mRNA expression in various glial cells and neurons in the hippocampus post-epileptogenesis.

In this study, we induced TLE in mice by stereotactically injecting 200 nl of kainic acid (KA; 1 ng/nl) into the CA1 region of the hippocampus at a rate of 1 nl/s. Following KA injection into the hippocampus, mice will experience an acute status epilepticus and subsequently enter a latent period lasting one to two weeks. During the ensuing period, the hippocampus of mice exhibits pathological changes similar to those observed in human HS and develop chronic spontaneous seizures. Therefore, we collected samples on the seventh day post-KA injection to investigate the potential mechanisms that contribute to epileptogenesis and HS [13]. The mice exhibited significant stage 5 seizures according to the modified Racine scale [14] following the injection and were subsequently maintained for 7 days before the collection of fresh hippocampal samples or preparation of whole-brain paraffin sections. These samples were subjected to single-cell RNA sequencing (ScRNA-seq), single-nucleus RNA sequencing (SnRNA-seq), and Xenium-based (mouse brain panel, 247 genes) spatial transcriptomics analyses. We analysed 31,390 glial cells and 48,221 neuronal nuclei from the ScRNA-seq and SnRNA-seq data, respectively. Differentially expressed genes (DEGs) between TLE and control hippocampi were identified, revealing significant transcriptomic alterations in various cell types 7 days post-TLE induction. Hundreds to thousands of mRNAs and their associated pathways showed dramatic changes. All 247 genes in the Xenium panel were detected in our mouse brain sections. Spatial clustering of these genes revealed 27 potential cell bodies and the spatial expression of major marker genes across the sections. Despite the limited number of genes in the panel, comparison of the DEGs between TLE and control hippocampi indicated significant glial responses and synaptic changes in neurons in mice with TLE.

By integrating the results from the three sequencing methods, we found that genes such as *Spp1*, *Trem2*, and *Cd68* were upregulated in both the Xenium data and four types of glial cells, while *Penk*, *Sorcs3*, and *Plekha2* were upregulated in both the Xenium data and two types of neurons. Conversely, *Tle4* and *Sipa1l3* were downregulated in both the Xenium data and four types of glial cells. In summary, in this study, a comprehensive single-cell transcriptome atlas of the hippocampus of mice with TLE was constructed; this atlas included glial cells, neurons, and high-resolution spatial maps. By delineating the pathological changes at the single-cell and spatial mRNA expression levels, we revealed inflammatory activation and altered cell interactions in the hippocampus in mice with TLE, and provide a foundational resource for further investigations into the mechanisms of HS and epileptogenesis.

## Materials and methods

### Animals

Eight-week-old C57BL/6 J male mice (weighing 20 ± 2 g) were purchased from *Vital River Laboratory Animals* (Beijing, China) and used in our study. All mice were housed at 22–25 °C and 50–60% humidity on a 12-h light/dark cycle and had access to food and water ad libitum. Our study was approved by the Ethics Committee of Xuanwu Hospital, and all animal procedures were performed in accordance with the guidelines provided by the Laboratory Animal Center of Capital Medical University Xuanwu Hospital.

### CA1 injection of KA and TLE induction

Half an hour before the initiation of the surgery, 2 mg/kg of Meloxicam (HY-B0261, MCE, United States) was administered subcutaneously. A 1% solution of 2,2,2-tribromoethanol (1 ml/100 g, T48402, Sigma-Aldrich, Germany) was injected intraperitoneally to anaesthetize the animals. For the mice in the TLE group, 200 nl of 1% KA (ab120100, Abcam, United States) was stereotaxically injected into the CA1 region of the hippocampus (AP -2.0 mm, ML -1.5 mm, DV -1.5 mm). Only mice with seizures that had a severity score (the Racine scale reached to or greater than 5) and lasted more than 30 min were considered to exhibit successful induction of TLE and were included in subsequent experiments. The control group mice were injected with an equivalent volume of sterile PBS. Seven days after TLE induction, the mice were anaesthetized, and after intracardial perfusion with ice-cold PBS (P1003, Solarbio, China) or PBS followed by paraformaldehyde (PFA; P110, Solarbio, China), the brains were collected for subsequent experiments.

### ScRNA-seq and SnRNA-seq data collection

ScRNA-seq and SnRNA-seq analyses were conducted by CapitalBio Technology (Beijing, China). After dissociation of entire ipsilateral hippocampus (with KA or PBS injection), single cells were sorted using microfluidic technology. The suspended cells were counted and subjected to quality control before being used to generate single-cell transcriptome libraries on the 10 × Genomics Chromium platform. Library construction was performed according to the manufacturer's instructions, and high-throughput sequencing was then performed on the Illumina NovaSeq 6000 platform. SnRNA-seq sample processing was conducted with a method similar to that used for ScRNA-seq sample processing. Tissue samples were mechanically and enzymatically dissociated, and the nuclei were isolated and purified. Subsequently, the suspended nuclei were counted and subjected to quality control prior to library construction for single-nucleus transcriptome analysis using the 10 × Genomics Chromium platform.

### Xenium-based spatial transcriptomics

For Xenium data collection, tissue samples were fixed with 4% PFA. The fixed brain tissue was then dehydrated through a series of graded ethanol solutions (E111991, Aladdin, China): 70% ethanol for 1 h, 80% ethanol for 1 h, 95% ethanol for 1 h, and 100% ethanol for 1 h. This was followed by immersion in xylene (X139941, Aladdin, China) and melted paraffin (YA0012, Solarbio, China). Subsequently, the tissue was embedded in melted paraffin, cooled, and sectioned into 5 μm slices using a microtome (HistoCore, Leica, Germany). Deparaffinization and rehydration were performed by immersing the sections in xylene and an ethanol gradient of decreasing concentrations, with each step lasting 10 min to prepare the sections for the Xenium procedure. Spatial transcriptome data were collected following the operational guidelines of the Xenium platform. Oligonucleotide probes complementary to the target mRNAs were synthesized and labelled with unique fluorescent tags. Tissue sections were then incubated with these probes under optimal conditions to ensure specific binding. To detect low-abundance transcripts, a signal amplification system, such as branched DNA technology, was used to enhance the fluorescence signals corresponding to the hybridized probes. High-resolution fluorescence microscopy was used to acquire images of the tissue sections. Multiple imaging rounds were performed to detect various fluorescence signals corresponding to different transcripts. The images were then stitched together to reconstruct the complete spatial map of the tissue section. The transcriptome data were sequenced with the spatial information, generating spatial transcriptome maps. Subsequently, the mouse brain sections were collected for Hematoxylin and Eosin (HE) (C0105M, Beyotime, China) staining, followed by image acquisition using a microscope (Mica, Leica, Germany).

### Bioinformatics analysis

The raw gene expression matrix was imported from the data files generated via the 10 × Genomics platform using the Read10X function in the Seurat package [15–19]. The imported data were used to create Seurat objects, ensuring that each gene was expressed in at least 3 cells and that at least 200 features were detected in each cell. Subsequently, the Seurat objects were subjected to initial quality control using the subset function to remove cells with fewer than 200 features or a percentage of mitochondrial genes greater than 10% in order to eliminate low-quality cells and potential cell debris. The DoubletFinder package was then used to detect and remove doublets. To address batch effects, batch correction was performed using the Harmony package. Highly variable genes were identified, principal component analysis (PCA) was performed using Seurat, and integration of multiple datasets was performed with Harmony to remove batch effects. Clustering analysis was performed using the FindNeighbors and FindClusters functions in Seurat, with the resolution parameter set to 0.2. Uniform manifold approximation and projection (UMAP) was used for dimensionality reduction and visualization, with the following parameters: dims = 1:50, n.epochs = 500, min.dist = 0.3, and spread = 1.

### Differential gene expression analysis

To identify genes with significant differential expression between different cell populations, differential gene expression analysis was performed using the FindAllMarkers function in the Seurat package [17] with the following parameters: test.use = "wilcox", min.pct = 0.1, and logfc.threshold = 0.1. The identified DEGs were further used for functional enrichment analysis as well as other relevant statistical analyses. The overlap of DEGs across the sequencing methods was visualized using UpSet plots generated with the VennDetail package (https://github.com/guokai8/VennDetail).

### Functional enrichment analysis

Functional enrichment analysis was conducted using the clusterProfiler package [20] by querying the Gene Ontology (GO) database to identify enriched pathways. GO analysis included the biological process (BP), cellular component (CC), and molecular function (MF) categories, with a significance threshold of *p* < 0.05. To visually display the results of differential gene expression analysis, volcano plots were generated using the EnhancedVolcano package, with a gene logFC threshold of ± 0.5 and a significance threshold of *p* < 0.05.

### Pseudotime analysis

Pseudotime analysis was performed using the Monocle3 package [21] to reveal the dynamic trajectories of cells in different states. First, ScRNA-seq data that had been preprocessed and subjected to quality control were transformed into a CellDataSet object. Then, the learn_graph function in Monocle3 was used to plot the cell trajectory, and the order_cells function was used to order the cells in pseudotime. The key parameter was set as follows: reduction_method = 'UMAP'. Our pseudotime analysis enabled the identification of cell differentiation trajectories and key branching points, furthering our understanding of the dynamic process of cell state transitions.

### Cell‒cell communication analysis

Cell‒cell communication analysis was conducted using the CellChat package [22] to delineate the signalling interactions and ligand–receptor interactions between different cell types. First, the preprocessed and quality-controlled ScRNA-seq data were imported into a CellChat object. Subsequently, a series of functions in the CellChat package were used for ligand‒receptor interaction analysis. The specific steps included identification of cell types and their marker genes, calculation of the probabilities of ligand‒receptor interactions between cells, analysis of signalling pathway enrichment, evaluation of the activity of each signalling pathway between different cell types, and construction of a cell–cell communication network to display the signalling interactions between different cell types. Our CellChat analysis enabled the identification of critical communication signalling between cells, revealing the interactions and functional coordination among different cell types in the tissue microenvironment.

### Gene set enrichment analysis (GSEA)

Differential expression genes were ranked by their average log fold change for downstream analysis. Gene sets from the Molecular Signatures Database (MSigDB.mm, version 2023.2) [23] were utilized, specifically focusing on all gene set except hallmark gene sets. We use GSEABase package (https://github.com/Bioconductor/GSEABase) ranked all DEG based on their log fold change from differential expression analysis. or each gene set, an enrichment score (ES) was calculated by walking down the ranked list, increasing a running-sum statistic when a gene was in the gene set and decreasing it when it was not. The statistical significance of the ES was determined using 1,000 permutations of the gene set labels. Nominal p-values were calculated for each gene set. False discovery rate (FDR) was controlled using the Benjamini–Hochberg procedure to adjust p-values. Significantly enriched gene sets (FDR < 0.25) were interpreted in the context of known biological processes and pathways. Visualization of GSEA results was performed using the clusterProfiler package in R [20].

## Results

### ScRNA-seq and SnRNA-seq analyses of TLE and control hippocampi

**Fig. 2:**
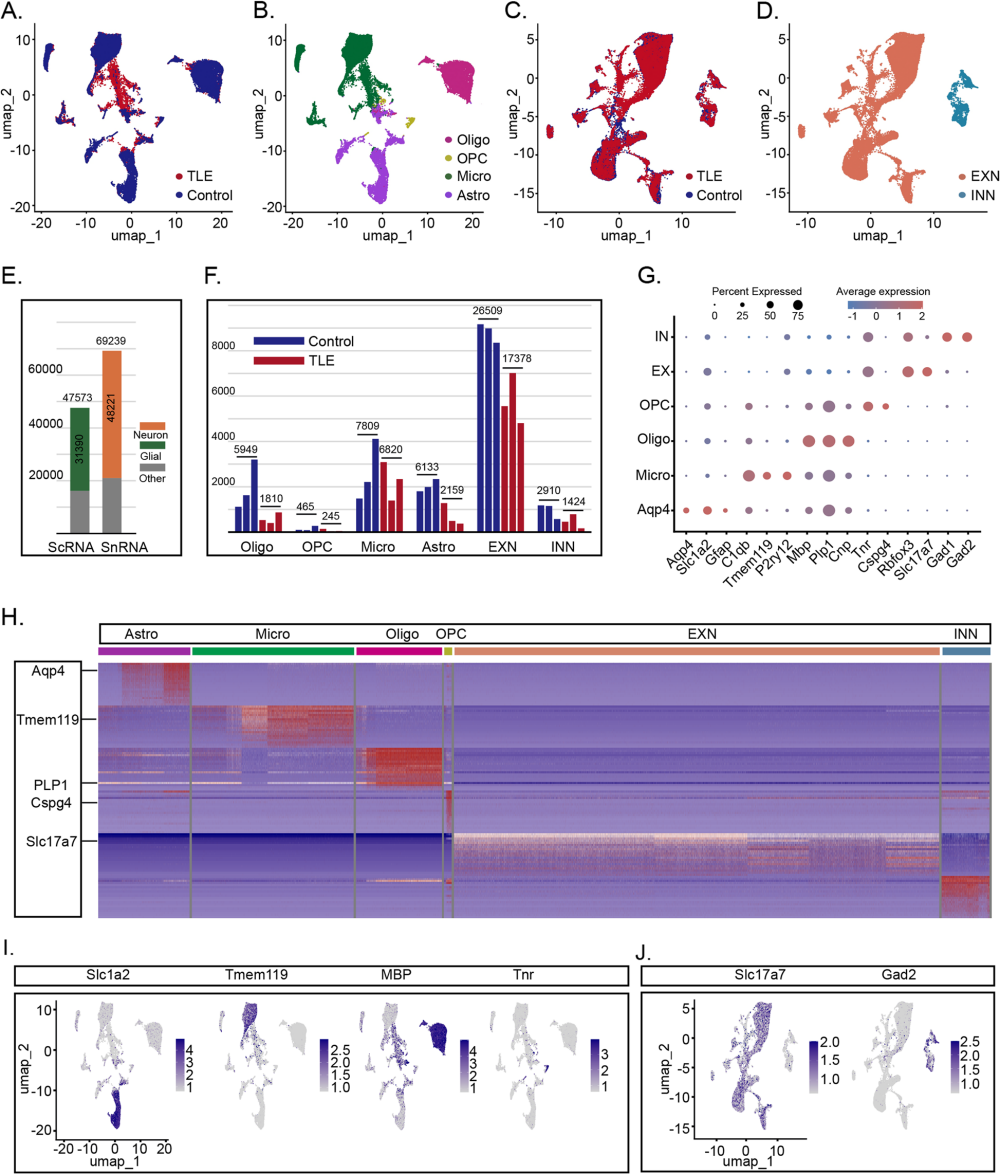
ScRNA-seq and SnRNA-seq Analyses of TLE and Control Hippocampus Samples. **A**-**B** UMAP plot of all glial cells in TLE and control hippocampi. **C**-**D** UMAP plot of all neurons in TLE and control hippocampi. **E** The green portion indicates the number of glial cells selected from the entire scRNA-seq dataset, while the orange portion represents the number of neurons selected from the entire snRNA-seq dataset. **F** Each bar represents an individual mouse, and the number labels indicating the total number of cells from three mice in the same group. **G** Expression levels of various marker genes used for the definition of cells in each cluster. **H** A combined gene expression heatmaps of glial cells from the ScRNA-seq and neurons from the SnRNA-seq data. **I**-**J** Representative UMAP plots showing the expression of marker genes in various glial cells and neurons

Six mice that developed significant status epilepticus (SE) following KA CA1 injection were selected for this study. The hippocampi of three mice with TLE and three control mice were subjected to ScRNA-seq analysis, while the hippocampi of another set of three mice with TLE and three control mice were subjected to SnRNA-seq analysis. This investigation yielded transcriptomic data from a total of 47,573 single cells and 69,239 single nuclei. Following dimensionality reduction and clustering using the UMAP algorithm, we selected 31,390 glial cells from ScRNA-seq and 48,221 neuronal cell nuclei from SnRNA-seq data for further analysis (Fig. 2E). The cell type distributions in the control and TLE groups were as follows: 5949 oligodendrocytes, 465 oligodendrocyte precursor cells (OPCs), 7809 microglia, 6133 astrocytes, 26,509 excitatory neurons (EXNs) and 2910 inhibitory neurons (INNs) in the control group and 1810 oligodendrocytes, 245 OPCs, 6820 microglia, 2159 astrocytes, 17,378 EXNs and 1424 INNs in the TLE group (Fig. 2F, Table S1). The following cell types were identified based on their specific marker genes (Fig. 2G): astrocytes (*Aqp4*, *Slc1a2*, *Gfap*), microglia (*C1qb*, *Tmem119*, *P2ry12*), oligodendrocytes (*Mbp*, *Plp1*, *Cnp*), OPCs (*Tnr*, *Cspg4*), EXNs (*Rbfox3*, *Slc17a7*), and INNs (*Gad1*, *Gad2*). UMAP plots were used to visualize the overall distribution of cells in both the TLE and control groups as well as the distribution of each cell type across the entire dataset (Fig. 2A-D). Additionally, we analysed the distributions of different marker genes within the overall sample set (Fig. 2I-J, Fig. S1A). A combined gene expression heatmaps of glial cells from the ScRNA-seq and neurons from the SnRNA-seq data were further generated to visualize significant transcriptional differences between various cell types (Fig. 2H; the full version is available in Fig. S1B).

### Transcriptomic alterations in mouse hippocampal glial cells and neurons following KA-induced TLE

**Fig. 3:**
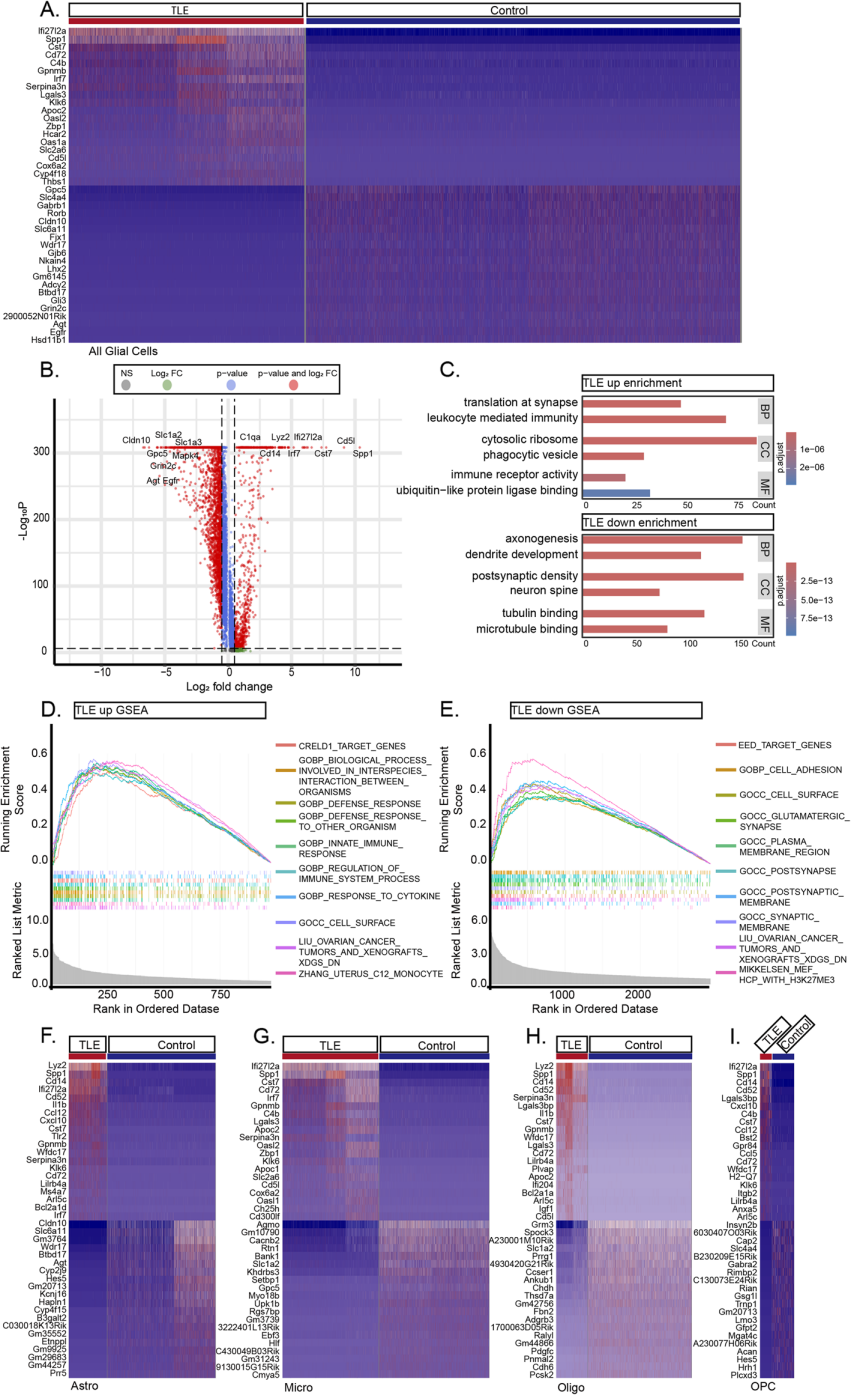
DEGs in Glial Cells Overall and Among Different Clusters. **A** Heatmap representation of the top 20 differentially expressed mRNAs in glial cells between TLE and control hippocampi. **B** Representative volcano plot showing the major DEGs in glial cells between TLE and control hippocampi. **C** GO enrichment analysis of the major upregulated and downregulated genes in the TLE group (BP: biological process; CC: cellular component; MF: molecular function). **D**-**E** GSEA analysis results for genes with elevated expression (**D**) and reduced expression (**E**) in glial cells of TLE group. **F**-**I** Representative heatmaps of the top 20 DEGs (*P* < 0.05 and Log_2_FC > 0.5) in astrocytes, microglia, oligodendrocytes, and OPCs in TLE and control hippocampi

Following KA-induced seizures, hippocampal glial cells in the mice exhibited profound transcriptional dysregulation leading to a widespread and intense stress response. Specifically, the expression levels of 834 genes, including *Spp1*, *Cd5l*, *Gpnmb*, *Cst7*, *Serpina3n*, and *Apoc2*, were elevated (*p* < 0.05 and Log_2_*FC* > 0.5) (Fig. 3A-B, Table S2). Conversely, 2939 genes, including *Slc6a11*, *Cldn10*, *Wdr17*, *Btbd17*, *Agt*, and *Slc4a4*, exhibited reduced expression. GO enrichment analysis of these DEGs revealed that those with elevated expression in glial cells from mice with TLE exhibited enrichment predominantly in pathways related to *translation at the synapse*, *leukocyte-mediated activity*, *phagocytic vesicles*, and *immune receptor activity*. In contrast, the genes with decreased expression were associated primarily with *axonogenesis*, *dendrite development*, *neuron spine formation*, and *tubulin binding* pathways (Fig. 3C). Similarly, the results of the GSEA analysis indicate that the genes upregulated in the TLE group are highly enriched in pathways such as *INNATE_IMMUNE_RESPONSE* and *RESPONSE_TO_CYTOKINE*. In contrast, the genes downregulated in the TLE group are primarily enriched in pathways including *GLUTAMATERGIC_SYNAPSE* and *POSTSYNAPTIC_MEMBRANE* (Fig. 3D-E). These findings suggest significant activation of immune- and inflammation-related pathways in glial cells, accompanied by widespread loss of supportive functions for neurons. Analysis of specific glial cell types further revealed that in mice with TLE, astrocytes exhibited upregulation of 830 genes and downregulation of 2538 genes, microglia showed upregulation of 845 genes and downregulation of 2790 genes, oligodendrocytes exhibited upregulation of 889 genes and downregulation of 2603 genes, and OPCs displayed upregulation of 234 genes and downregulation of 2735 genes. The expression of proinflammatory genes, such as *Spp1*, *Lyz2*, and *Cd14*, was increased in each glial cell type, indicating intense inflammatory activation. GO enrichment and GSEA analysis also indicated the activation of inflammatory pathways and a reduction in or complete loss of neurosupportive capacity across these glial cells (Fig. 3F-I, Fig. S2A-D, Tables S3-6).

To determine the intrinsic heterogeneity driving glial cells towards this proinflammatory transition, pseudotime analysis was performed. The results showed a stark contrast in the distribution of glial cells between the TLE and control groups on the UMAP plots. Glial cells from the control group were located predominantly at the starting points of trajectories, while glial cells from the TLE groups were concentrated at the endpoints, displaying centripetal convergence on the UMAP plots (Fig. S3A-C). This pattern indicates that the extensive inflammatory phenotypic transformation is accompanied by increased transcriptional homogeneity among various glial cells. However, whether this transition is driven primarily by intrinsic mechanisms or intercellular interactions remains unclear.

**Fig. 4:**
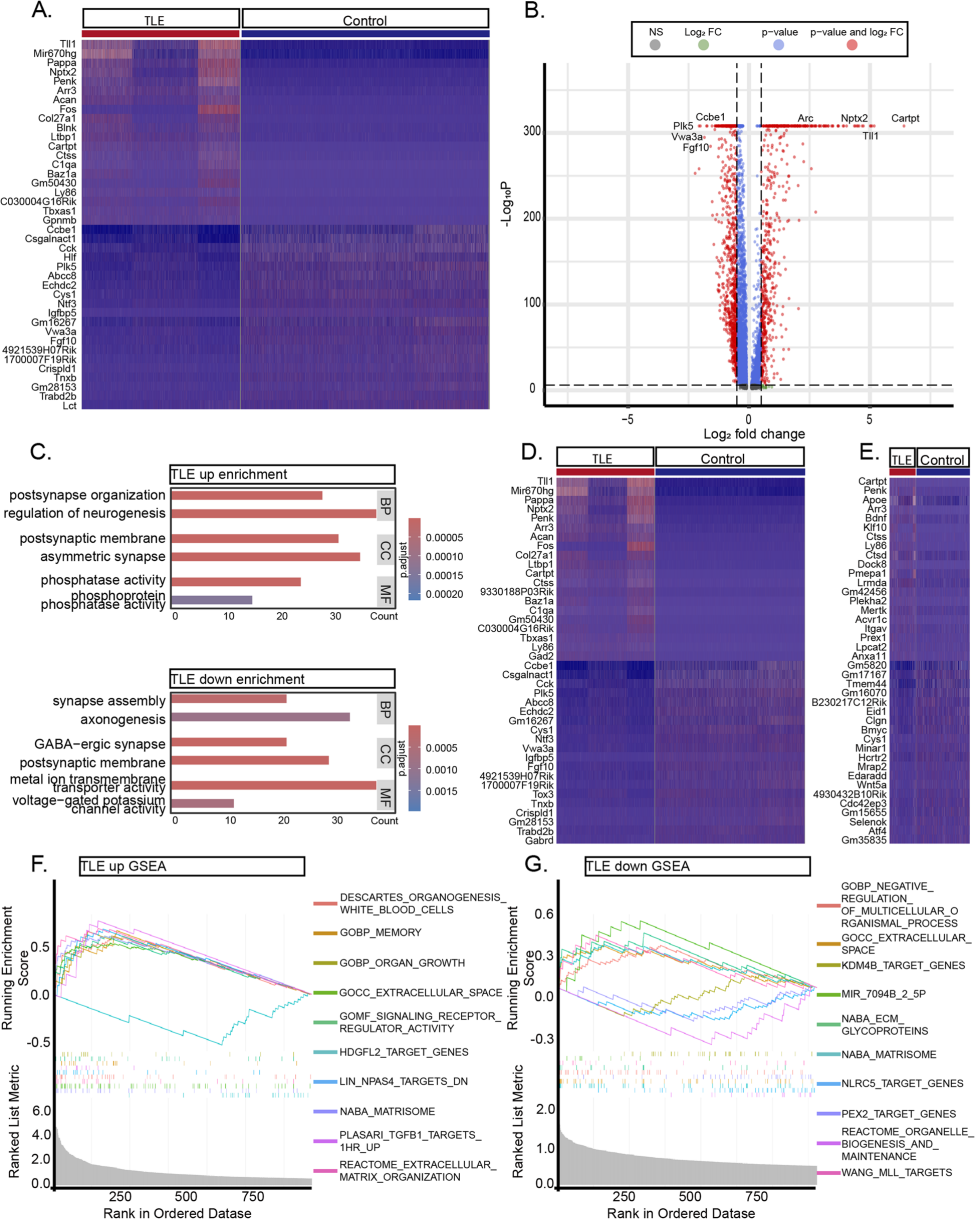
DEGs in Overall Neurons, EXNs, and INNs. **A** Heatmap representation of the top 20 differentially expressed mRNAs in neurons between TLE and control hippocampi. **B** Representative volcano plot of the major DEGs in neurons between TLE and control hippocampi. **C** GO enrichment analysis of the major upregulated and downregulated genes in the TLE group. **D**-**E** Representative heatmaps of the top 20 DEGs in EXNs and INNs in TLE and control hippocampi. **F**-**G**: GSEA analysis results for genes with elevated expression (**F**) and reduced expression (**G**) in neurons of TLE group

Due to limitations in obtaining neuronal transcriptomic data solely via ScRNA-seq, we performed SnRNA-seq on hippocampal tissues from the same batch of mice. Differential gene expression analysis of all neurons revealed elevated expression of 671 genes (identified by *p* < 0.05 and Log_2_*FC* > 0.5), including *Cartpt*, *Acan*, *Tll1*, *Ly86*, *Pappa*, and *Gm50430* (Fig. 4A-B, Table S7), and reduced expression of 942 genes, including *Plk5*, *Ntf3*, *Vwa3a*, *Cys1*, *Igfbp5*, and *Fgf10*. GO enrichment analysis indicated that the genes with elevated expression in neurons from the TLE group exhibited significant enrichment in pathways such as *postsynapse organization*, r*egulation of neurogenesis*, *asymmetric synapse formation*, and *phosphate activity*. In contrast, the downregulated genes exhibited enrichment in *synapse assembly*, *axonogenesis*, *GABAergic synapse formation*, and *voltage-gated potassium channel activity* (Fig. 4C). Otherwise, we conducted GSEA analysis and found that neurons in the TLE group are most significantly enriched in functional categories such as *MEMORY* and *ORGAN_GROWTH* (Fig. 4F). In contrast, neurons in the control group predominantly retain pathways related to *ECM_GLYCOPROTEINS* and *EXTRACELLULAR_SPACE* (Fig. 4G). Then, we analysed specific neuronal cell types, finding 723 upregulated and 1052 downregulated genes in EXNs and 91 upregulated and 103 downregulated genes in INNs. The primary DEGs for each neuronal cell type as well as the corresponding results of GO enrichment and GSEA analysis are detailed in Fig. 4D-E, Fig. S4A-B, and Tables S8-9.

### Cellular communication analysis based on glial and neuronal transcriptomic data

**Fig. 5:**
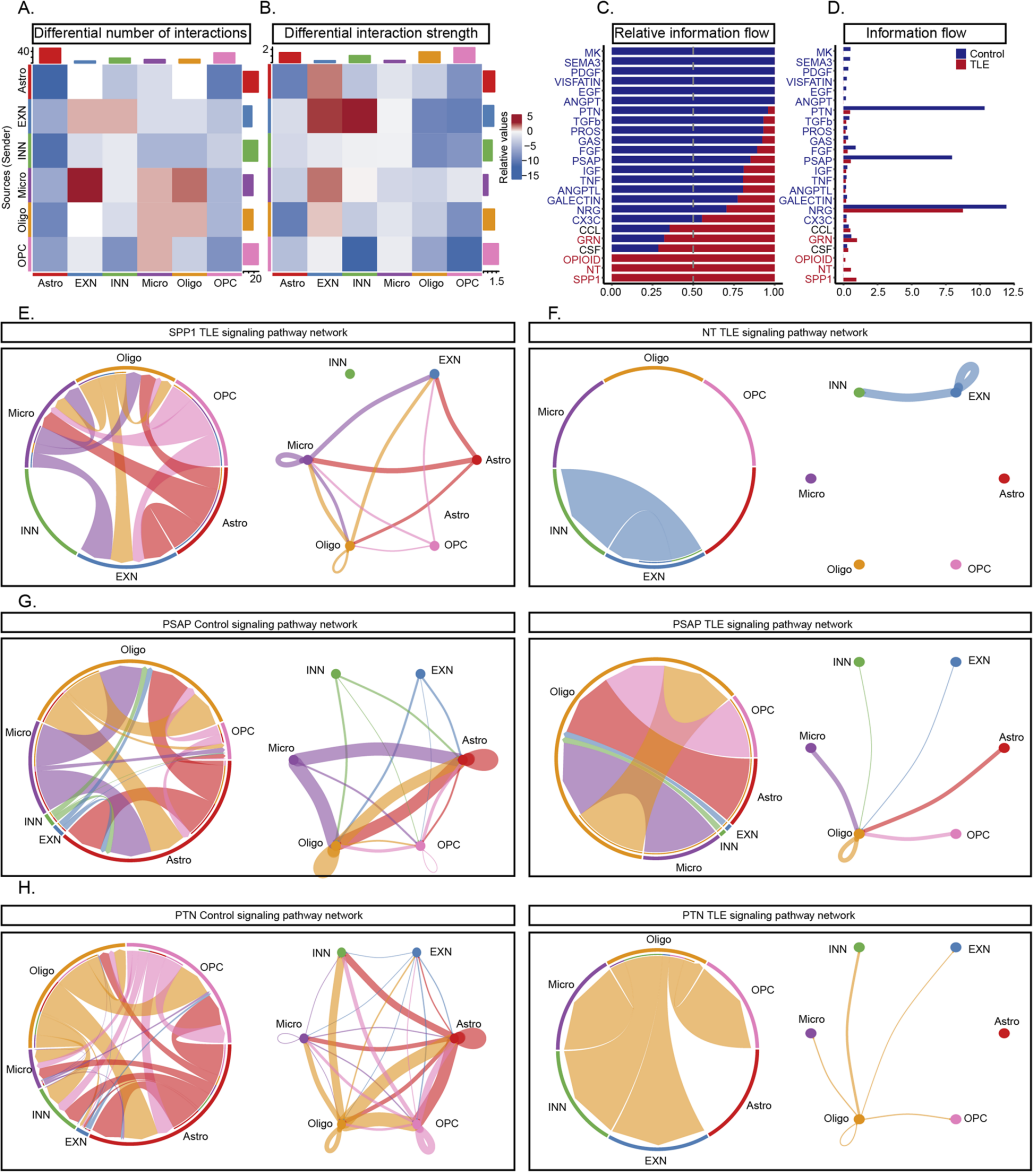
Changes in Interactions Between Cells in the TLE Hippocampus, as Revealed by ScRNA-seq and SnRNA-seq. **A** Differential numbers of interactions within each cluster in the TLE and control hippocampus. **B** Differential interaction strengths within each cluster of the TLE and control hippocampus. **C**-**D** Relative information flow of the altered cell communication events. **E**–**F** Interactions mediated via the Spp1 and NT signalling pathway were significantly activated only in the TLE hippocampus. In the circle plots and chord diagrams, the thickness of the connecting lines between different clusters represents the strength of the correlation. No interactions mediated via these two pathways were observed in the control group. **G**-**H** Interactions mediated via the Psap and Ptn signalling pathway exhibited significant differences between TLE and control hippocampi. For the Pasp pathway, the complexity of interactions between different cell types was significantly reduced. For example, the output from microglia to astrocytes and the interactions within astrocytes were notably decreased (**G**). For the Ptn pathway, only the output from oligodendrocytes to other cells remained active in the TLE group (**H**)

Based on the results of transcriptomic analysis of glial cells and neurons, we performed a cellular communication analysis using the CellChat package. By integrating the transcriptomic data of neurons and glial cells, we evaluated the mRNA levels of numerous cytokines and their receptors across various cell types. We specifically detailed the cytokine–receptor pairs that showed significant changes in the TLE group. Notably, hippocampal EXNs and microglia, followed by oligodendrocytes and microglia, exhibited the most significant changes in the number of interactions (Fig. 5A). Although the number of altered interactions among neurons (between EXNs and INNs or among EXNs themselves) was lower than that among glial cells, these interactions showed the greatest changes in strength (Fig. 5B). Next, we analysed the specific signalling pathways enriched in these altered cellular interactions and their differences between the control and TLE groups. In the TLE group, the intercellular interaction pathways with the most significant increases in communication strength were the *Spp1* and *NT* pathways, whereas the *PTN* and *PASP* pathways exhibited the most significant decreases in communication strength (Fig. 5C-D). The communication strength of the *Spp1* pathway, which was significantly increased in the TLE group, was particularly notable. This pathway was almost completely inactive in the hippocampus in the control group but became highly active post-seizure, with various cells acting as both senders and receivers, thus forming an intricate network of interactions among different glial cells and EXNs (Fig. 5E, Fig. S5A). Similarly, the activity of the *NT* pathway, which is composed primarily of BDNF and its receptors and downstream genes, was increased. The upstream mediator *BDNF* was expressed mainly in EXNs in the TLE group, while its receptors were distributed predominantly among INNs and EXNs (Fig. 5F, Fig. S5B). Conversely, the *Psap* and *Ptn* pathways presented a more complex interaction profile but were generally suppressed in the TLE group, with simplified interaction networks. Specifically, a significant decrease in receptor levels in the *Psap* pathway was observed in the TLE group, while the decrease in *Ptn* pathway activity was mediated mainly by the almost complete loss of *Ptn* expression in astrocytes and OPCs (Fig. 5G-H, Fig. S5C-D). In summary, the intercellular interactions within the hippocampi of mice with TLE exhibited alterations consistent with the transcriptomic alterations. These changes may indicate a shift in glial cell function from providing support to promoting damage, possibly driven by overactive interactions among neurons and leading to excessive synchronization.

### Xenium-based spatial transcriptomic analysis of TLE and control mouse brain sections

The KA CA1 injection-induced seizure model is a model of focal epilepsy. However, neither the ScRNA-seq data of glial cells nor the SnRNA-seq data of neurons can be used to elucidate the spatial distribution and cellular localization of mRNA transcripts within the damaged and relatively normal regions of the TLE hippocampus. To address this limitation, we performed Xenium-based spatial transcriptomic sequencing on two paraffin-embedded coronal sections containing the dorsal hippocampus (one from the TLE group and one from the control group). We detected a total of 245,925 spots (131,833 from TLE slice and 114,092 from Control slice), and by this approach we determined the expression levels of all 247 genes in the mouse brain panel across all 5-μm spots.

**Fig. 6:**
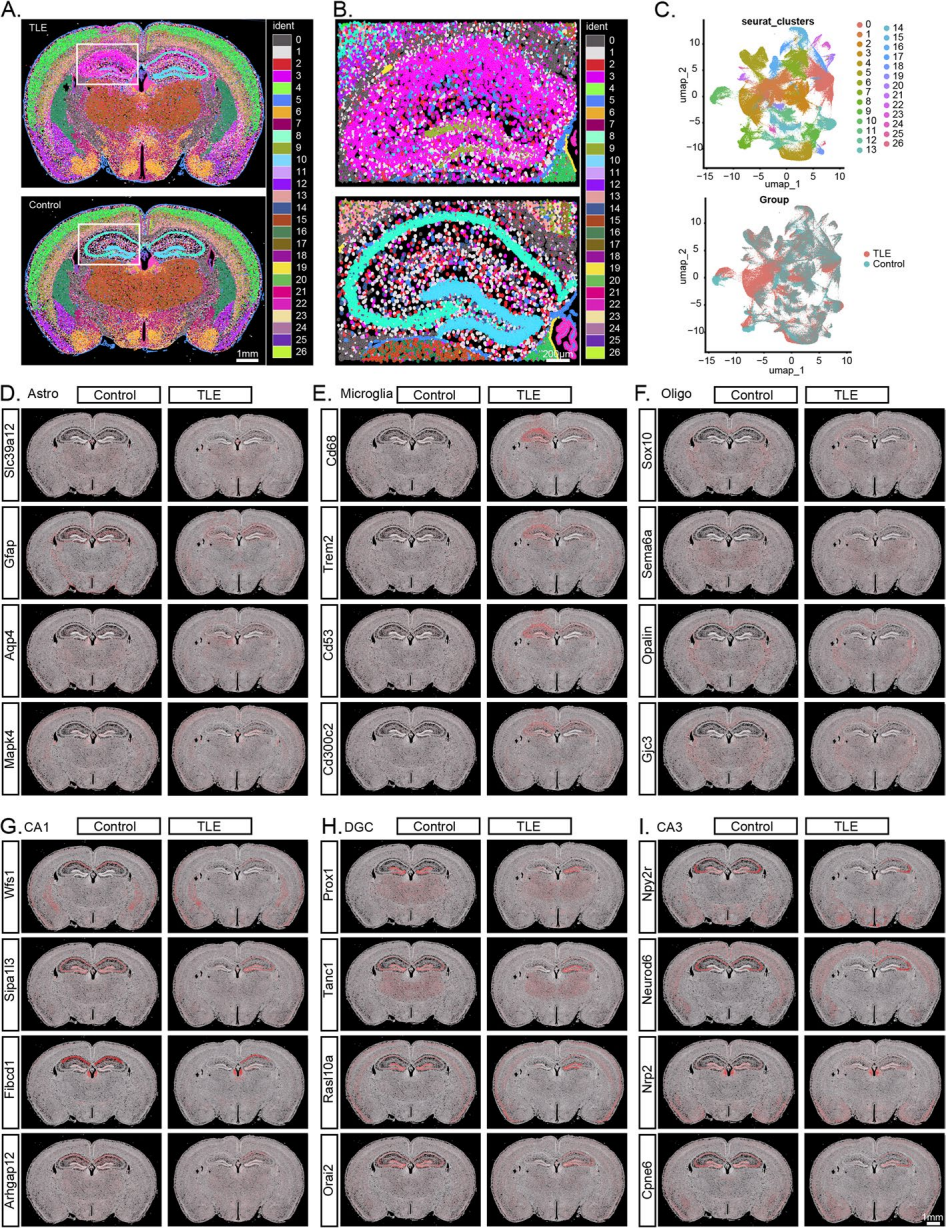
Xenium-Based Spatial Transcriptomics Analysis of TLE and Control Mouse Brain Sections. **A** Whole-brain transcriptomic information obtained through Xenium-based spatial transcriptomics analysis, with reconstructed cell bodies displayed in different colours. The white box highlights the TLE and control hippocampal regions. **B** Enlarged view of the hippocampal region highlighted in A. **C** UMAP plot showing the 27 identified cell types. **D**-**I** High-resolution expression patterns of marker genes for astrocytes, microglia, oligodendrocytes, CA1 pyramidal neurons, dentate gyrus granule cells, and CA3 pyramidal neurons in Control and TLE sections. The left side represents the brain of a control mouse, while the right side represents the brain of a TLE mouse. Each red dot represents a positive expression point for a gene, with a diameter of 5 μm

Based on the spatial clustering results for these genes, we reconstructed the somatic presence of 27 cell types and delineated their spatial distribution features across both entire sections and specific hippocampal regions in the TLE and control groups (Fig. 6A). We designated regions of interest (ROIs) in the KA-injected epileptic hippocampus and the control hippocampus for further analysis. Within these ROIs, all 27 cell types were reconstructed, but the cellular composition shifted dramatically, showing significant morphological differences and transcriptomic heterogeneity between the TLE and control hippocampi (Fig. 6B, Table S11). Additionally, we performed dimensionality reduction clustering based on the gene expression levels of each somatic cell body and visualized the distribution in a UMAP plot (Fig. 6C).

In the control mouse brain section, we demonstrated the high-resolution spatial distributions of different marker genes used to define the primary cell types (Fig. 6D-I): astrocytes (*Slc39a12*, *Gfap*, *Aqp4*, *Mapk4*), microglia (*Cd68*, *Trem2*, *Cd53*, *Cd300c2*), oligodendrocytes (*Sox10*, *Sema6a*, *Opalin*, *Gjc3*), CA1 pyramidal neurons (*Wfs1*, *Sipa1l3*, *Fibcd1*, *Arhgap12*), dentate granule cells (*Prox1*, *Tanc1*, *Rasl10a*, *Orial2*), and CA3 pyramidal neurons (*NPY2r*, *Neurod6*, *Nrp2*, *Cpne6*). The detailed spatial expression profiles of other marker genes (e.g., *Syndig1* and *Strip2*) are provided in Fig. S6. It is evident that the expression levels of many marker genes show significant differences between the ipsilateral hippocampi of control and TLE mice, as well as between the ipsilateral and contralateral hippocampi of TLE mice.

**Fig. 7:**
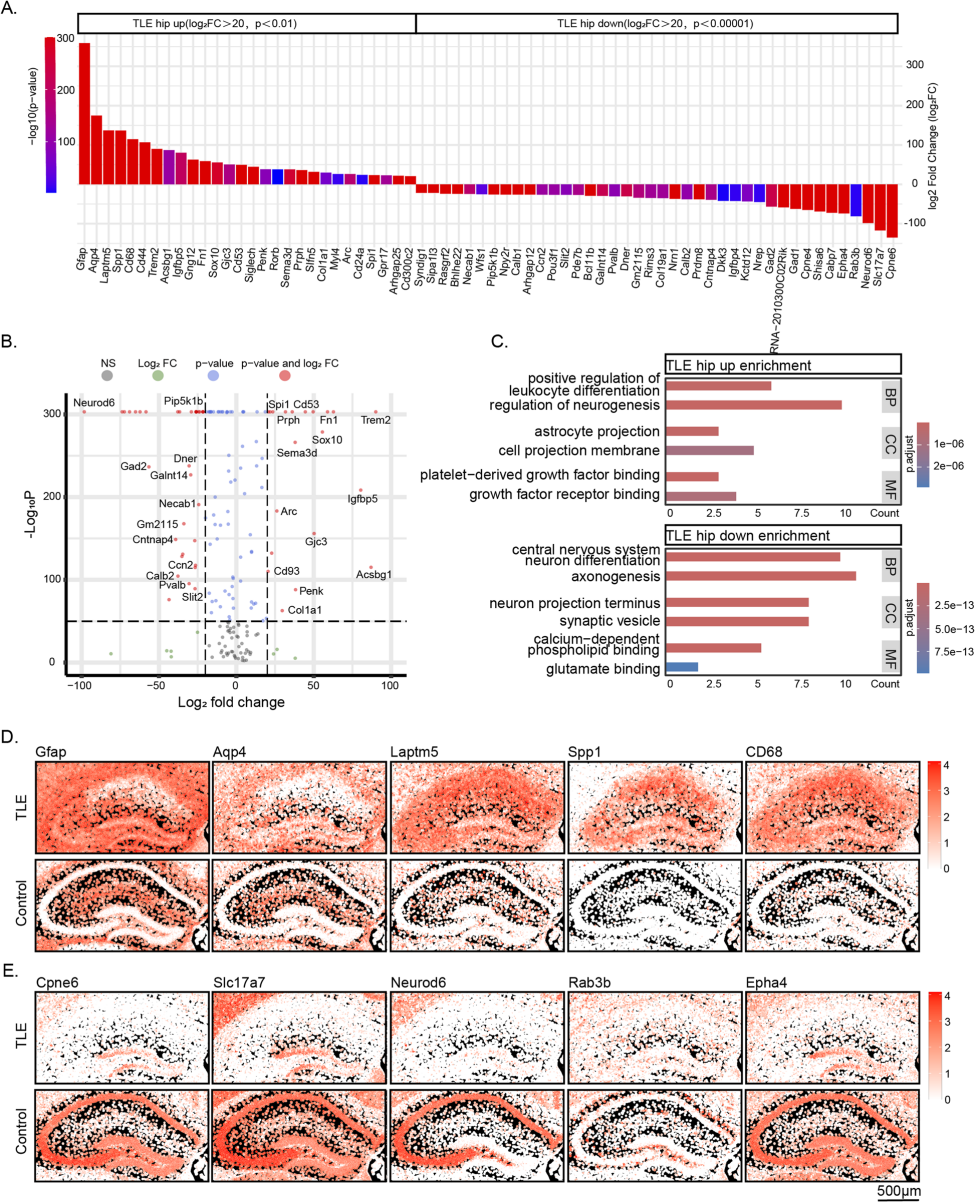
DEGs Between TLE and Control Hippocampi, as Revealed via the Xenium Platform. **A** The top 28 upregulated mRNAs and the top 40 downregulated mRNAs in the TLE hippocampus identified in the Xenium data (*P* < 0.01 and Log_2_FC > 20). **B** Representative volcano plot of the differentially expressed genes in the hippocampus. **C** GO enrichment analysis based on the differentially expressed genes between TLE and control hippocampi. **D** High-resolution spatial distribution of the top 5 upregulated mRNAs (Gfap, Aqp4, Laptm5, Spp1, and Cd68) in the TLE hippocampus. **E** High-resolution spatial distribution of the top 5 downregulated mRNAs (Cpne6, Slc17a7, Neurod6, Rab3b, and Epha4) in the TLE hippocampus

We then compared the genes exhibiting significant changes in the TLE hippocampus within the Xenium ROIs. The spatial variation in mRNA expression in the Xenium data was significantly more pronounced than that in the ScRNA-seq and SnRNA-seq data. We listed the top 28 genes with increased expression and the top 40 genes with decreased expression (identified by *p* < 0.01 and Log_2_FC > 20) (Fig. 7A). The main genes with increased expression included *Gfap*, *Aqp4*, *Laptm5*, *Spp1*, and *Cd68*, while those with decreased expression included *Cpne6*, *Slc17a7*, *Neurod6*, *Rab3b*, and *Epha4* (Fig. 7A-B).

Enrichment analysis based on these genes indicated the activation of pathways such as *positive regulation of leukocyte differentiation* and *astrocyte projection* in the TLE hippocampus, consistent with the reactive changes in microglia and astrocytes. Conversely, pathways such as *axonogenesis*, *neuron projection terminus*, and *synaptic vesicle function* were inhibited. To visually compare the differential gene expression between the TLE and control hippocampi, we generated high-resolution spatial maps of these DEGs across various subregions of the hippocampus in both groups (Fig. 7D-E, Fig. S7-S8). The DEGs enriched in glial cells exhibited obvious diffuse intergroup differences across the entire hippocampus, whereas the genes enriched in neurons displayed comparatively spatially specific alterations within different hippocampal subregions. These genes could thus be significantly associated with reactive glial proliferation and neuronal reduction.

### Integrated differential analysis of xenium, ScRNA-seq, and SnRNA-seq data

**Fig. 8:**
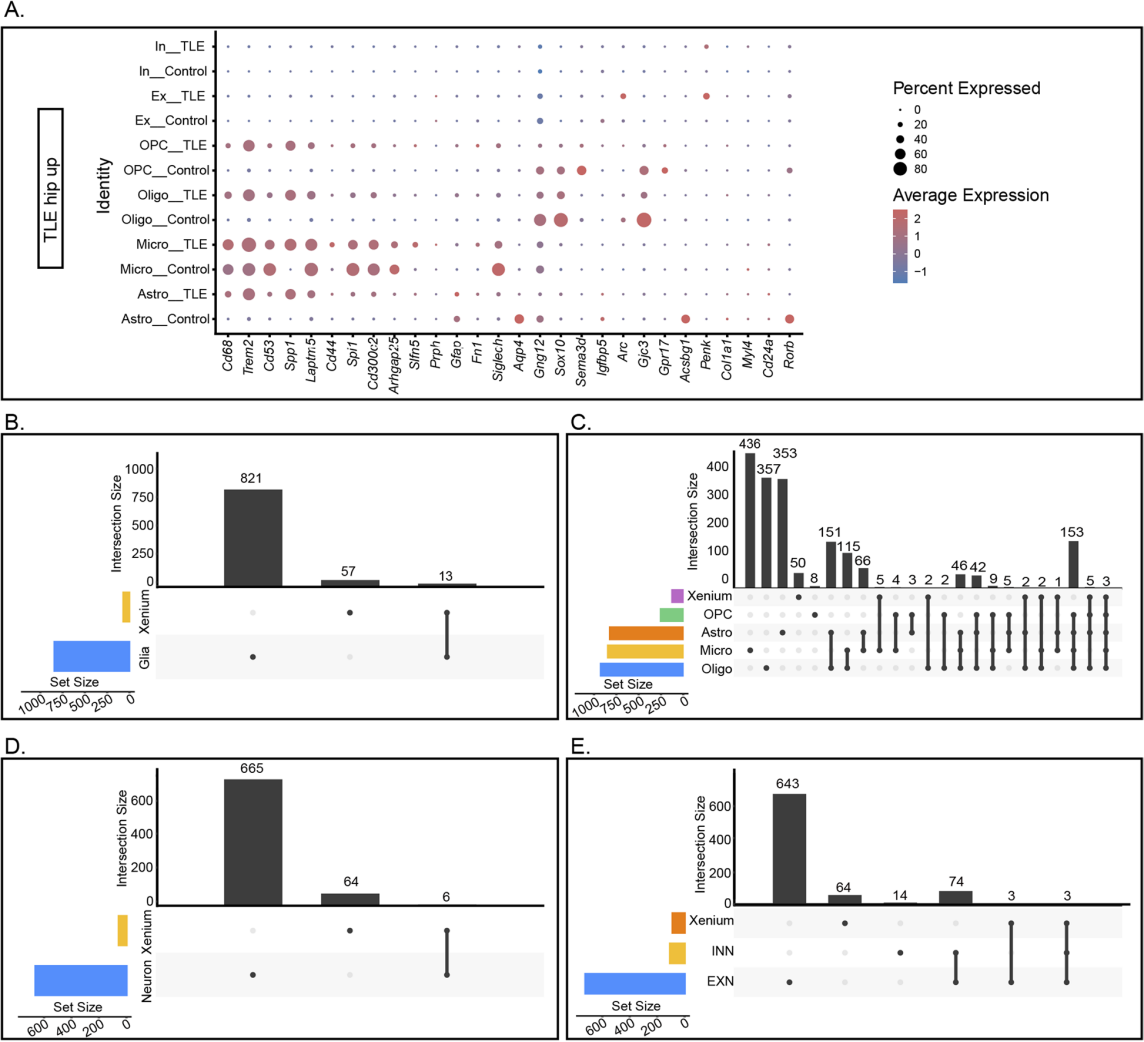
Analysis of Genes Identified as Upregulated in Both the Xenium Data and the ScRNA-seq/SnRNA-seq Data. **A** Distribution of the top 28 upregulated genes identified in the Xenium data among various glial cells and neurons. **B**-**C** Correlation of genes upregulated in the Xenium data with similar trends in overall glial cells and specific clusters. The colour bars on the horizontal axis represent the DEGs between the groups for the corresponding clusters, while the grey bars indicate the cluster-specific DEGs associated with the black dots. **D**-**E** Correlations of genes identified as upregulated in the Xenium data with similar trends in overall neurons, EXNs, and INNs

**Fig. 9:**
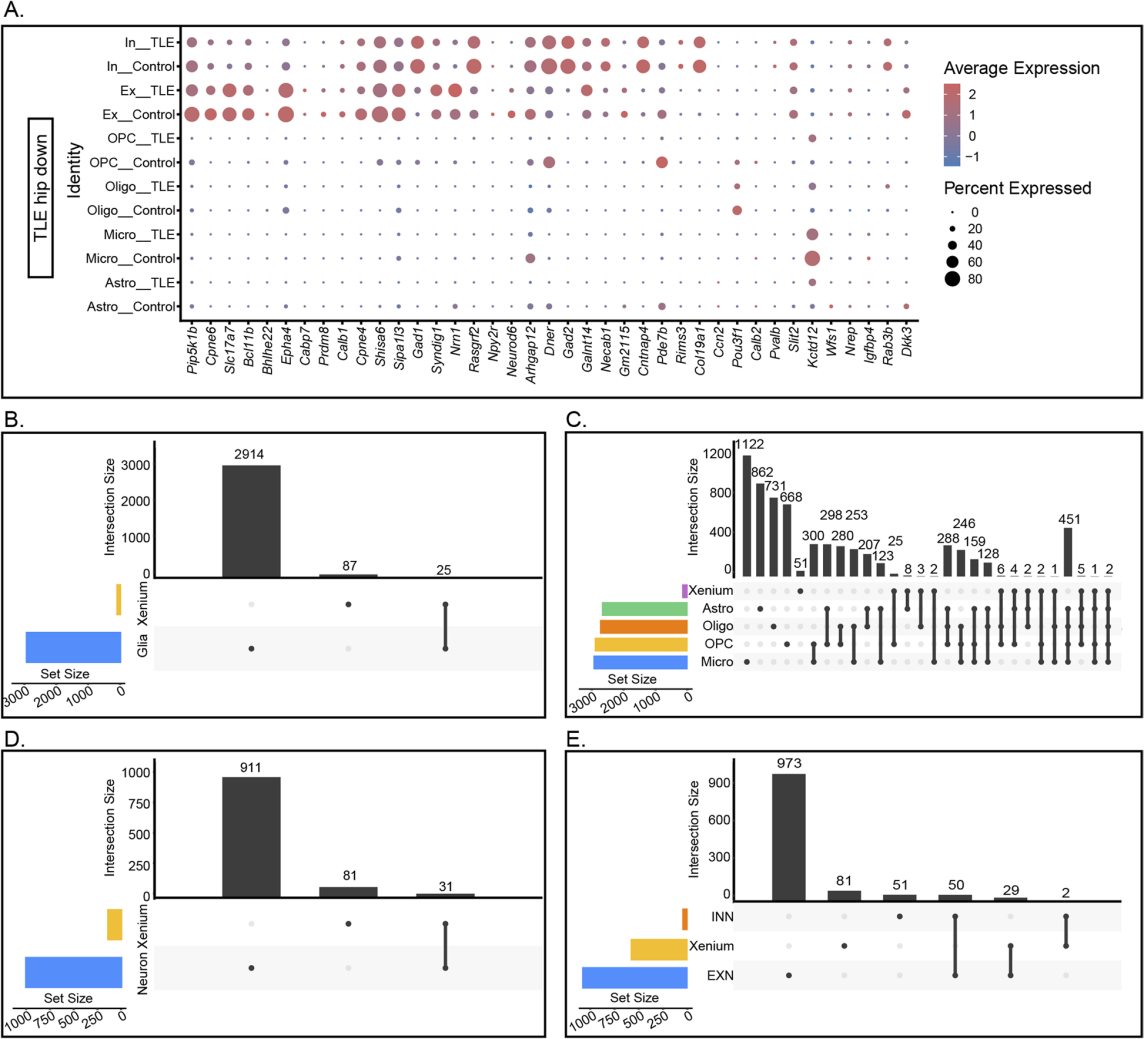
Analysis of Genes Downregulated in Both the Xenium Data and the ScRNA-seq/SnRNA-seq Data. **A** Distribution of the top 40 downregulated genes identified in the Xenium data among various glial cells and neurons. **B**-**C** Correlation of genes identified as downregulated in the Xenium data with similar trends in overall glial cells and specific clusters. **D**-**E** Correlations of genes identified as downregulated in the Xenium data with similar trends in overall neurons, EXNs, and INNs

The DEGs identified in the Xenium data could not be used for true spatial single-cell mapping. Therefore, we examined the expression distribution of the DEGs identified in the Xenium data across various cell types based on the ScRNA-seq and SnRNA-seq data (Fig. 8A, Fig. 9A). The 28 upregulated genes were primarily distributed among different types of glial cells, whereas the 40 downregulated genes were expressed found in neurons. However, many genes exhibited expression trends different from those in the Xenium data.

To identify key changes in the hippocampus of mice with TLE, we integrated data from the Xenium, ScRNA-seq, and SnRNA-seq analyses to compare the common and unique DEGs across different cell types and spatial transcriptomes (criteria: *p* < 0.05 and Log_2_*FC* > 0.5). Analysis of these data in conjunction with glial cell types revealed that 57 genes were uniquely upregulated in the Xenium data, 821 genes were uniquely upregulated in glial cells, and 13 genes were upregulated in both the Xenium data and glial cells. Further classification of glial cells indicated that 50 genes were uniquely upregulated in the Xenium data, whereas 353, 436, 357, and 8 genes were uniquely upregulated in astrocytes, microglia, oligodendrocytes, and OPCs, respectively. Additionally, 153 genes were upregulated in all four glial cell types, with only *Spp1*, *Trem2*, and *Cd68* upregulated in both the Xenium data and the four types of glial cells (Fig. 8B-C).

By integrating the spatial transcriptomic data with sequencing data from neurons, we found that 64 genes were uniquely upregulated in the Xenium data, 665 genes were uniquely upregulated in neurons, and 6 genes were upregulated in both the Xenium data and neurons. Further classification of neurons revealed that 64 genes were uniquely upregulated in the Xenium data, whereas 643 and 14 genes were uniquely upregulated in EXNs and INNs, respectively. Additionally, 74 genes were upregulated in both neuron subtypes, with only *Penk*, *Sorcs3*, and *Plekha2* upregulated in both the Xenium data and these two neuron subtypes (Fig. 8D-E). The specificity of the downregulated genes in the hippocampus of mice with TLE is shown in Fig. 9. According to our analysis, only *Tle4* and *Sipa1l3* were downregulated in both the Xenium data and all four types of glial cells, and no genes were downregulated in both the Xenium data and the two neuron subsets.

## Discussion

In this study, we employed both ScRNA-seq and SnRNA-seq technologies to simultaneously profile the hippocampal transcriptomes of glial cells and neurons in mice with TLE. Additionally, we utilized a Xenium-based spatial transcriptomics approach to obtain high-resolution RNA localization information in the hippocampus. Consistent with previous reports, our findings indicate widespread activation of inflammatory responses in glial cells within the hippocampus in mice with TLE [11, 24, 25]. Specifically, microglia and astrocytes exhibited a tendency to shift towards an overactive disease-associated phenotype. Furthermore, oligodendrocytes demonstrated a significant proinflammatory transcriptomic shift post-seizure. These phenotypic changes in glial cells were visualized through pseudotime analysis, revealing centripetal convergence of their transcriptomes in response to seizure events. These inflammatory phenotypes of oligodendrocytes have attracted increasing attention, yet whether these changes are driven primarily by endogenous pathway activation or through interactions with other cell types remains unclear, and further mechanistic studies are required. Our SnRNA-seq analysis of neurons revealed an increase in the assembly of excitatory asymmetric synapses in the hippocampus in mice with TLE, accompanied by suppression of GABAergic synapses. This alteration is crucial for subsequent seizure events [26, 27]. Despite the overall neuronal damage and loss, enrichment analysis based on the DEGs indicated the occurrence of reactive neurogenesis in the hippocampus, which has complex impacts on hippocampal repair and epileptic circuit formation. For example, Ledergerber et al. reported a transient increase in cell proliferation in the subgranular zone shortly after KA injection, followed by significant impairment of neurogenesis by day 14 [28]. Nevertheless, another study demonstrated that the hippocampal stem cell niche of mice was disrupted by day 8 following KA intervention [29]. Additionally, a study on the hippocampi of human patients indicated that longer durations of epilepsy are associated with a sharp decline in neurogenesis [30]. These findings support the notion that hippocampal neurogenesis in the context of TLE-HS undergoes a dynamic evolution, characterized by initial activation followed by impairment. The correlation between newly generated neurons and seizure activity varies at different stages of this process.

Unexpectedly, although the number of INNs in the TLE hippocampus was significantly decreased post-induction, the transcriptomic profile of INNs was relatively stable compared to that of other cell types, with 91 transcripts significantly upregulated and 103 downregulated. The vulnerability and functional decline of inhibitory interneurons in TLE patients are key foci in research on epileptogenic mechanisms and seizure inhibition, yet our results highlight a predominant role for EXNs in the changes occurring post-TLE induction [31, 32]. The possibility of targeting both cell types to restore the excitatory–inhibitory balance requires careful consideration. In addition to the significant reduction in neurons (attributable to damage resulting from KA-induced neuronal hyperactivity), our sequencing data revealed a lower number of glial cells in the TLE group than in the control group, in contrast to reports of gliosis in the TLE hippocampus [6]. This may be due to the fact that the hippocampi of TLE mice are under intense stress, robust cellular response and inflammatory activities affecting cellular integrity. As a result, these cells exhibited greater fragility during sample preparation and sequencing experiments compared to the control group, leading to the acquisition of fewer cells or nuclei in the TLE group. Cell‒cell communication analysis based on the DEGs also indicated that glial cells in the TLE hippocampus seemed to lose their supportive functions for neurons, instead displaying a phenotype that promoted damage, while communication between EXNs and INNs was excessively activated. This finding suggests that KA injection may induce drastic phenotypic transformations in glial cells, leading to excessive synchronization of local hippocampal circuits.

Generally, ScRNA-seq provides a comprehensive transcriptomic profile of glial cells but fails to capture neuronal data. Conversely, SnRNA-seq can obtain transcriptomic information from neuronal nuclei but sacrifices a substantial amount of glial cell data. Therefore, to achieve a more complete transcriptomic profile, we employed both ScRNA-seq and SnRNA-seq on hippocampal tissues from the same batch of mice to simultaneously analyse glial cells and neurons. Otherwise, we performed high-resolution spatial transcriptomics via the Xenium platform, which provides unprecedented spatial resolution at the single-cell or subcellular level, revealing intricate cell‒cell interactions within complex tissues. Considering that the ScRNA-seq and SnRNA-seq samples included both the KA injection site (dorsal CA1) and the entire remaining hippocampus, the results may be diluted by changes outside the core seizure focus. In contrast, the Xenium-Based Spatial Transcriptomics data selectively analyzed the dorsal hippocampus region, minimizing potential interference from other hippocampal areas and revealing greater differences at the mRNA level. Therefore, in our analysis, DEGs for ScRNA-seq and SnRNA-seq were identified using a threshold of *p* < 0.05 and Log_2_FC > 0.5, while for Xenium-Based Spatial Transcriptomics, a more stringent threshold of *p* < 0.01 and Log_2_FC > 20 was applied. This higher threshold for Xenium-Based Spatial Transcriptomics was chosen to ensure the robustness of the identified DEGs. Additionally, comparing the trends across different sequencing methods provided increased confidence in the DEGs identified.

Our aim was to map the precise distributions of neurons and glial cells in the TLE hippocampus to understand the mechanisms underlying the organization of and functional changes in the local neural network. Our data revealed near-single-cell-level spatial expression patterns and post-TLE expression changes in genes across various hippocampal subregions, providing valuable insights into the roles and mechanisms of these genes in hippocampal function and epileptic network formation. Generally, the genes identified as upregulated in the Xenium data were expressed primarily in glial cells, while the downregulated genes were expressed primarily in neurons. This trimodal sequencing approach revealed a diffuse stress-induced phenotypic shift in hippocampal glial cells, especially astrocytes and microglia, with neurons exhibiting more spatially specific transcriptomic alterations. Notably, the Xenium data demonstrated a high detection rate of DEGs between TLE and control hippocampi. In our integrated analysis, many genes identified in the Xenium data showed consistent changes in the transcriptomes of glial cells and neurons. Specifically, the *Spp1*, *Trem2*, and *Cd68* genes were significantly upregulated across various glial cell types according to both the Xenium and single-cell transcriptomic data. The activation of pathways associated with these genes indicates a prominent state of inflammatory activation in the hippocampus [33–35]. The roles of these genes in epilepsy or other neurological disorders have recently attracted attention from several research teams. For instance, a recent study by Che et al. utilized bulk RNA sequencing on human samples and found that *SPP1* levels were significantly elevated in the hippocampus of TLE-HS patients. They also suggested that the *SOX2-OT/miR-671-5p/SPP1* axis may be a potential therapeutic target for TLE-HS [36]. Another study on Alzheimer's disease indicated that S*pp1* derived from perivascular macrophages promotes microglial phagocytosis of synapses. This finding offers a valuable reference for investigating the potential mechanisms by which microglia-derived *Spp1* may influence seizure activity in our study [37]. In addition, *Trem2*, as a significant receptor for *Apoe*, has been a focal point in the field of neurodegenerative disease research. Recent reports have indicated that lipid-accumulated reactive astrocytes (LARAs) can influence seizure activity by *Apoe*-dependently transferring abnormal lipid droplets within neurons in both TLE-HS patients and animal models. However, it remains unclear whether *Trem2* is involved in this process. [38, 39]. This observation suggests that persistent inflammation, which is predominantly mediated by glial cells, may play a critical role in the development of TLE-HS.

However, our study has clear limitations. First, the hippocampal damage and epileptic seizure in human TLE-HS patients form a mutually reinforcing process with a complex and still unclear pathogenesis [40]. The KA-induced epilepsy model represents acute hippocampal excitotoxic injury, was employed in this study to investigate the mechanisms underlying epileptogenesis. However, this model cannot fully replicate the actual pathological conditions of clinical TLE-HS. The seven-day postinjection period is considered a latent phase with minimal spontaneous seizures [41]; thus, our findings provide insights into hippocampal damage and epileptogenic mechanisms post-SE but cannot explain the initiation of TLE. Our Xenium-based spatial transcriptomics analysis was limited to the KA injection site (including the dorsal dentate gyrus); thus, data from other hippocampal regions covered by the ScRNA-seq and SnRNA-seq analyses are lacking, resulting in inconsistencies in differential gene expression trends. Additionally, the limited number of genes in the Xenium mouse brain panel resulted in the identification of fewer DEGs than via ScRNA-seq and SnRNA-seq. Despite its ability to generate high-resolution spatial transcriptomic data, the Xenium platform cannot localize all spots to original cells, delineating only simulated cell body contours through mRNA clustering, and this mapping falls short of the precise spatial mapping achieved via ScRNA-seq and SnRNA-seq. In neuronal studies, we categorized neurons into EXNs and INNs for a broader overview of hippocampal neuronal damage, an approach that does not consider the intrinsic transcriptomic differences among neurons in different hippocampal subregions. Given the spatial heterogeneity of KA-induced hippocampal damage, this simplification helps to exclude intrinsic differences among hippocampal neurons, facilitating the effective exploration of pathogenic transcriptomic changes.

## Conclusion

In summary, we integrated single-cell, single-nucleus, and Xenium spatial transcriptomics approaches to construct a high-resolution, cell-specific transcriptome atlas of the hippocampus in mice with TLE, encompassing mRNA data for various glial cells and neurons and achieving near-single-cell resolution spatial mapping. Our atlas revealed inflammatory activation and altered cell interactions in the hippocampus in mice with TLE and constitutes a reliable database for further exploration of HS and epileptogenic mechanisms.

## Supplementary Information

Supplementary Material 1: Figure S1. A. Representative UMAP plots showing other marker genes. B. Complete version of the combined gene expression heatmaps of glial cells from the ScRNA-seq and neurons from the SnRNA-seq data of the top 20 DEGs across all clusters.

Supplementary Material 2: Figure S2. A-D. Volcano plots of major DEGs and the corresponding Gene Ontology (GO) enrichment analysis results for various glial cells. From top to bottom, the results are shown for astrocytes, microglia, oligodendrocytes, and OPCs. From left to right are the volcano plots of DEGs, GO enrichment analysis results, and GSEA analysis results.

Supplementary Material 3: Figure S3. Trajectories of various glial cell clusters predicted using Monocle. A represents the results for all glial cells, B represents glial cells from the control group, and C represents glial cells from the TLE group.

Supplementary Material 4: Figure S4. A-B. Volcano plots of major DEGs and the corresponding GO enrichment analysis results in excitatory and inhibitory neurons. From left to right are the volcano plots of DEGs, GO enrichment analysis results, and GSEA analysis results.

Supplementary Material 5: Figure S5. Changes in the expression of Spp1, Bdnf, Psap, Ptn, and their receptors and downstream genes in each cluster in TLE and control hippocampi. A, C. The expression of Spp1 and Psap is elevated to varying degrees in astrocytes, microglia, oligodendrocytes, and OPCs within the TLE group. The Spp1 signaling pathway in TLE microglia and the Psap signaling pathway in both astrocytes and oligodendrocytes exhibited more classic activation states (as indicated by the red boxes). B. Bdnf is predominantly upregulated in excitatory neurons in the TLE group (as indicated by the blue boxes). D. The expression of Ptn is decreased to varying degrees in astrocytes, oligodendrocytes, and OPCs in the TLE group. In the control group, the Ptn signaling pathway was primarily active in microglia and OPCs (indicated by blue boxes).

Supplementary Material 6: Figure S6. A-B. Representative HE staining results of mouse brain sections from the Control group (A) and TLE group (B). The upper images display the whole brain at low magnification, while the lower images provide a high magnification view of the boxed region, specifically showing the ipsilateral hippocampus. C. High-resolution spatial expression distribution of the major marker genes used to define various neurons in control mouse brain sections. For each gene, the left side represents the Control group, while the right side represents the TLE group.

Supplementary Material 7: Figure S7. A. Spatial expression distribution of the top 28 upregulated genes in ipsilateral hippocampus of TLE and control mouse.

Supplementary Material 8: Figure S8. A. Spatial expression distribution of the top 40 downregulated genes in ipsilateral hippocampus of TLE and control mouse.

Supplementary Material 9.

Supplementary Material 10.

Supplementary Material 11.

Supplementary Material 12.

Supplementary Material 13.

Supplementary Material 14.

Supplementary Material 15.

Supplementary Material 16.

Supplementary Material 17.

Supplementary Material 18.

Supplementary Material 19.

## Data Availability

Data is provided within the manuscript or supplementary information files.
