# Supplementary figures and images for: Single-cell, single-nucleus and xenium-based spatial transcriptomics analyses reveal inflammatory activation and altered cell interactions in the hippocampus in mice with temporal lobe epilepsy
Source: Biomark Res. 2024 Sep 13;12:103. doi: 10.1186/s40364-024-00636-3 (PMC11396644; doi:10.1186/s40364-024-00636-3)

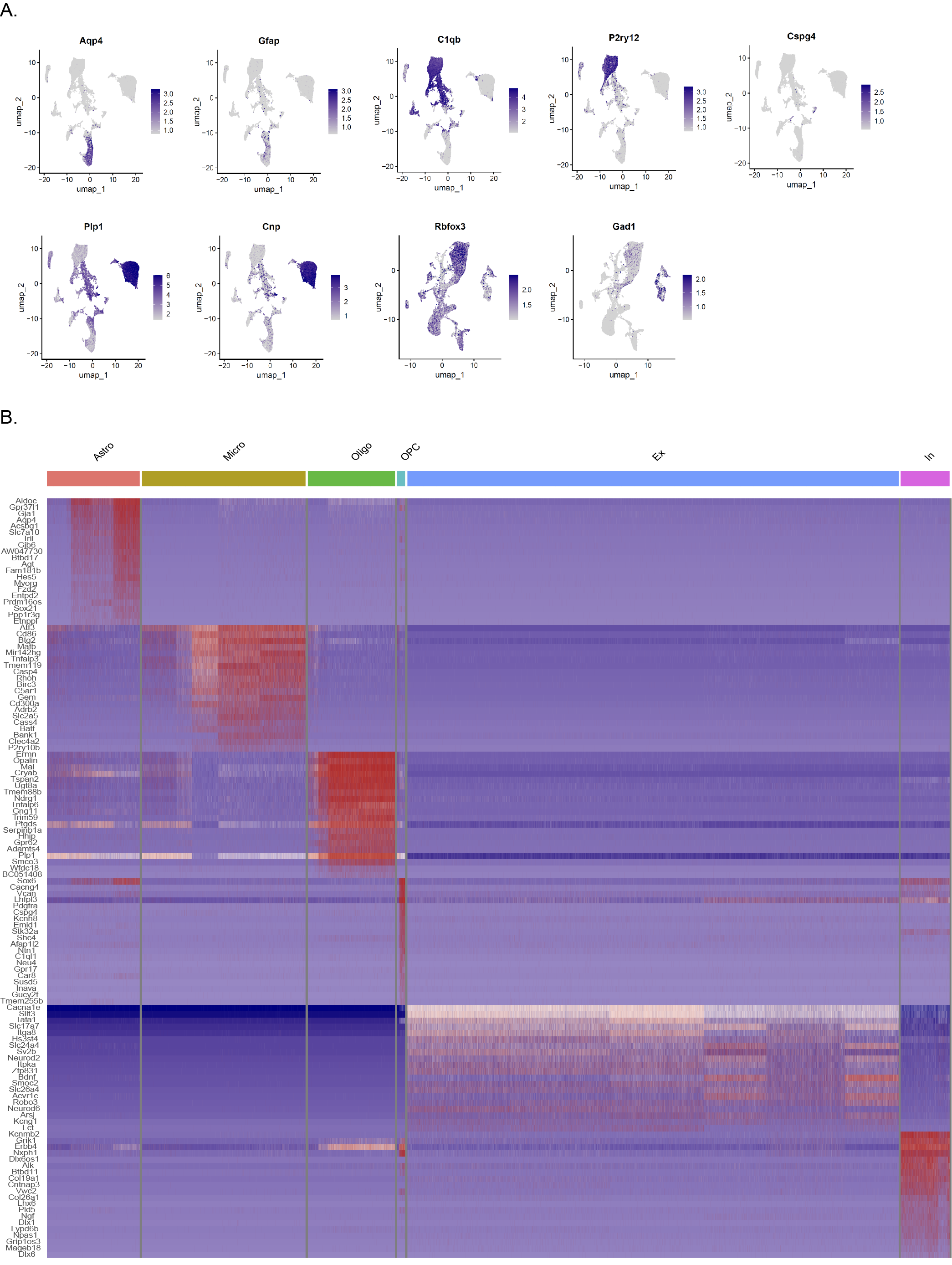

Supplement: Supplementary file 1 — Supplementary Material 1: Figure S1. A. Representative UMAP plots showing other marker genes. B. Complete version of the combined gene expression heatmaps of glial cells from the ScRNA-seq and neurons from the SnRNA-seq data of the top 20 DEGs across all clusters. [file 40364_2024_636_MOESM1_ESM.png]

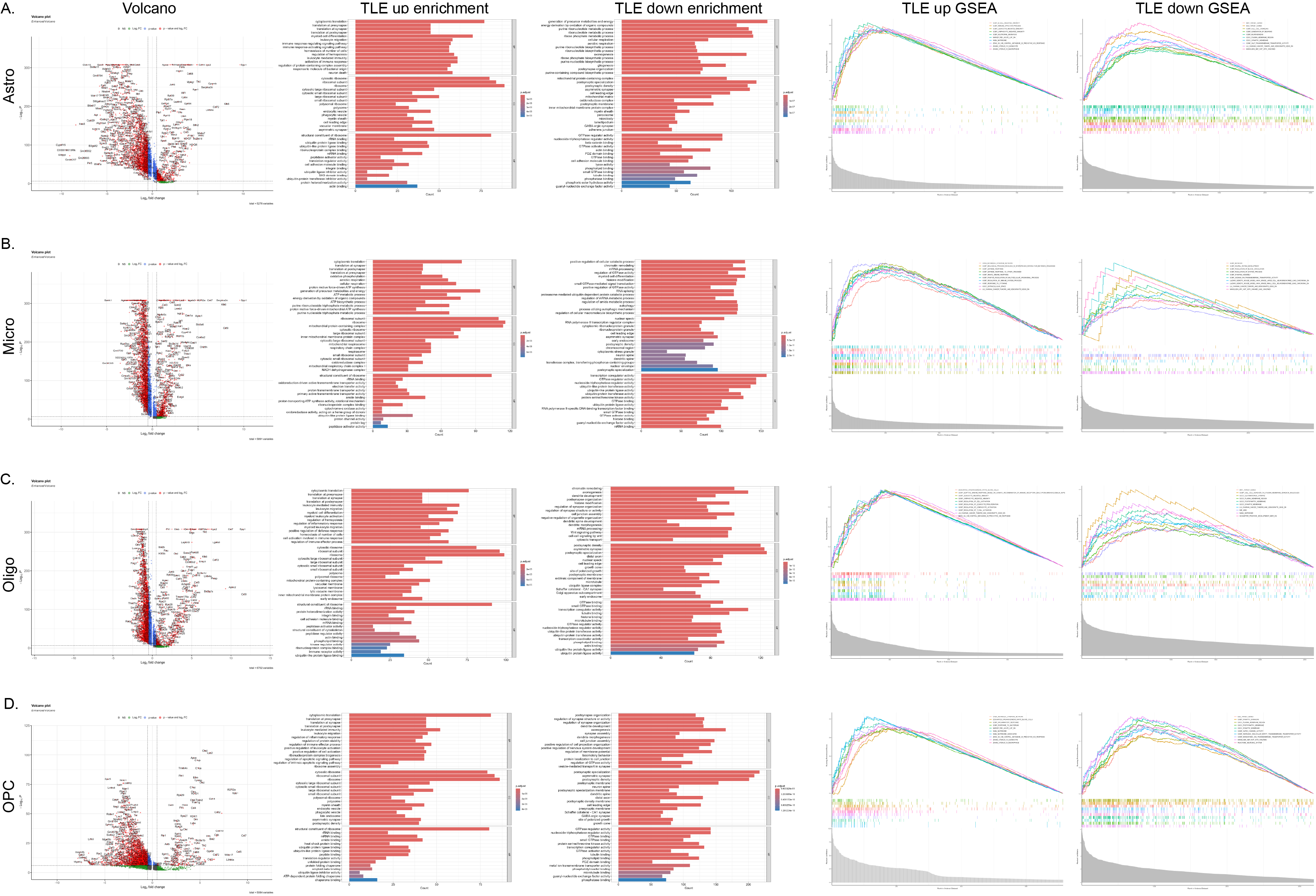

Supplement: Supplementary file 2 — Supplementary Material 2: Figure S2. A-D. Volcano plots of major DEGs and the corresponding Gene Ontology (GO) enrichment analysis results for various glial cells. From top to bottom, the results are shown for astrocytes, microglia, oligodendrocytes, and OPCs. From left to right are the volcano plots of DEGs, GO enrichment analysis results, and GSEA analysis results. [file 40364_2024_636_MOESM2_ESM.png]

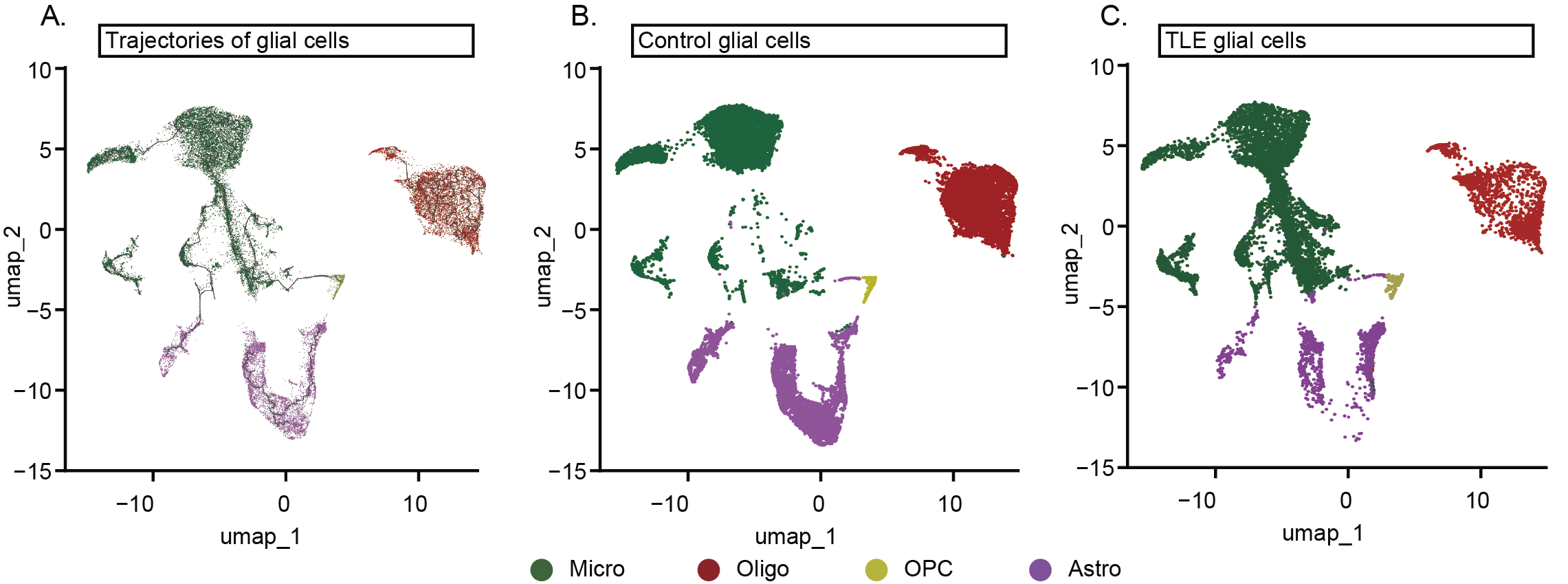

Supplement: Supplementary file 3 — Supplementary Material 3: Figure S3. Trajectories of various glial cell clusters predicted using Monocle. A represents the results for all glial cells, B represents glial cells from the control group, and C represents glial cells from the TLE group. [file 40364_2024_636_MOESM3_ESM.png]

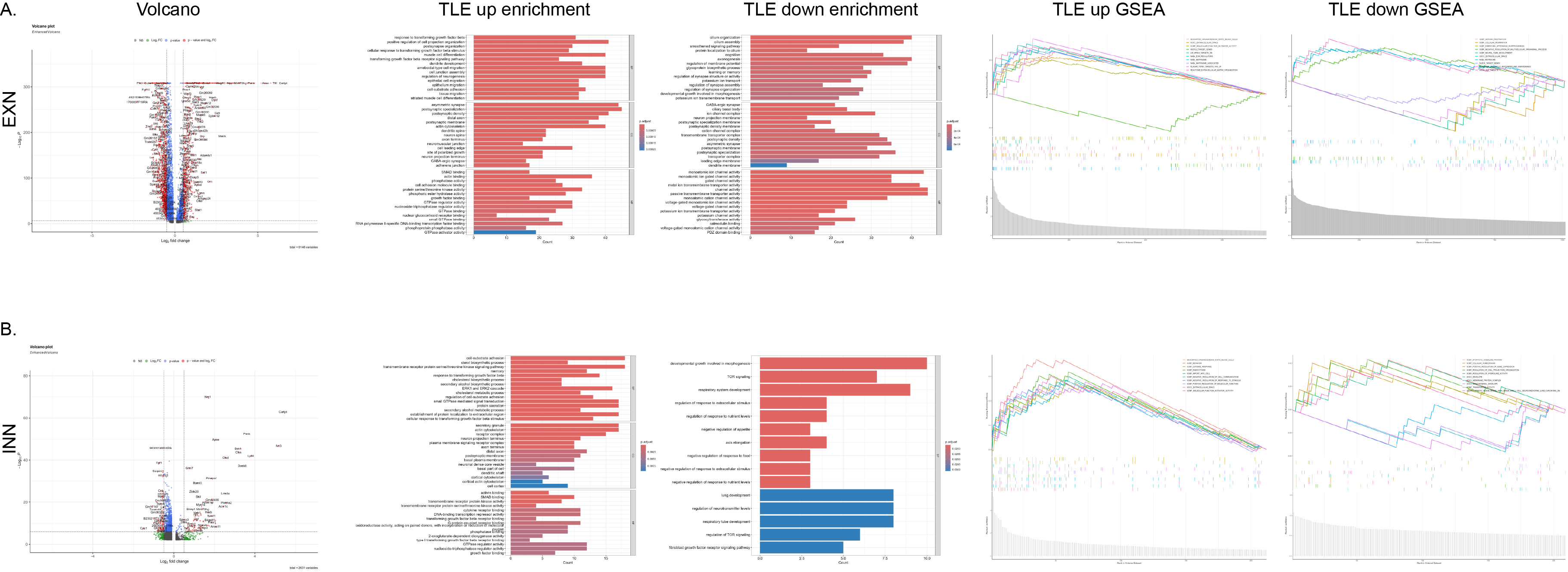

Supplement: Supplementary file 4 — Supplementary Material 4: Figure S4. A-B. Volcano plots of major DEGs and the corresponding GO enrichment analysis results in excitatory and inhibitory neurons. From left to right are the volcano plots of DEGs, GO enrichment analysis results, and GSEA analysis results. [file 40364_2024_636_MOESM4_ESM.png]

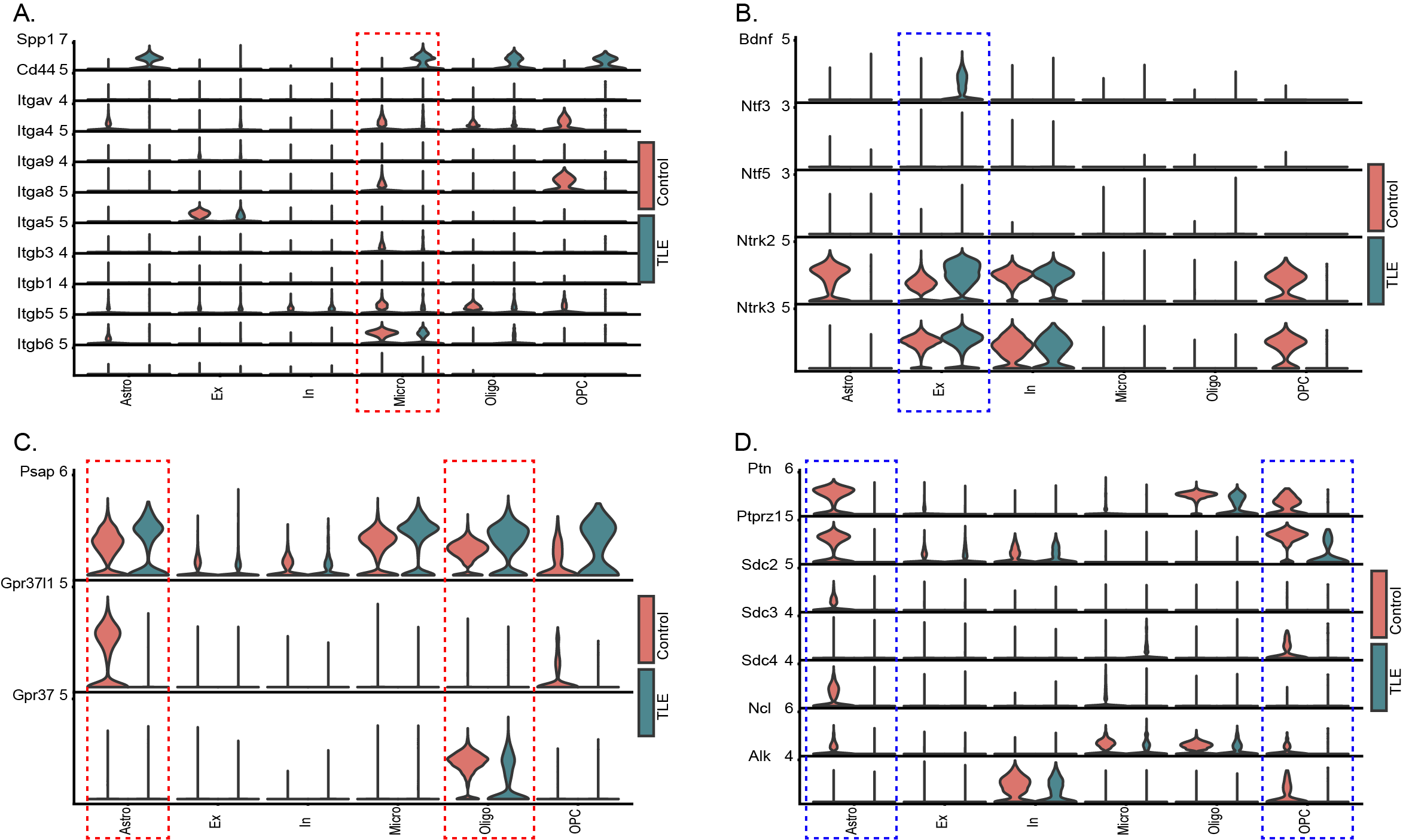

Supplement: Supplementary file 5 — Supplementary Material 5: Figure S5. Changes in the expression of Spp1, Bdnf, Psap, Ptn, and their receptors and downstream genes in each cluster in TLE and control hippocampi. A, C. The expression of Spp1 and Psap is elevated to varying degrees in astrocytes, microglia, oligodendrocytes, and OPCs within the TLE group. The Spp1 signaling pathway in TLE microglia and the Psap signaling pathway in both astrocytes and oligodendrocytes exhibited more classic activation states (as indicated by the red boxes). B. Bdnf is predominantly upregulated in excitatory neurons in the TLE group (as indicated by the blue boxes). D. The expression of Ptn is decreased to varying degrees in astrocytes, oligodendrocytes, and OPCs in the TLE group. In the control group, the Ptn signaling pathway was primarily active in microglia and OPCs (indicated by blue boxes). [file 40364_2024_636_MOESM5_ESM.png]

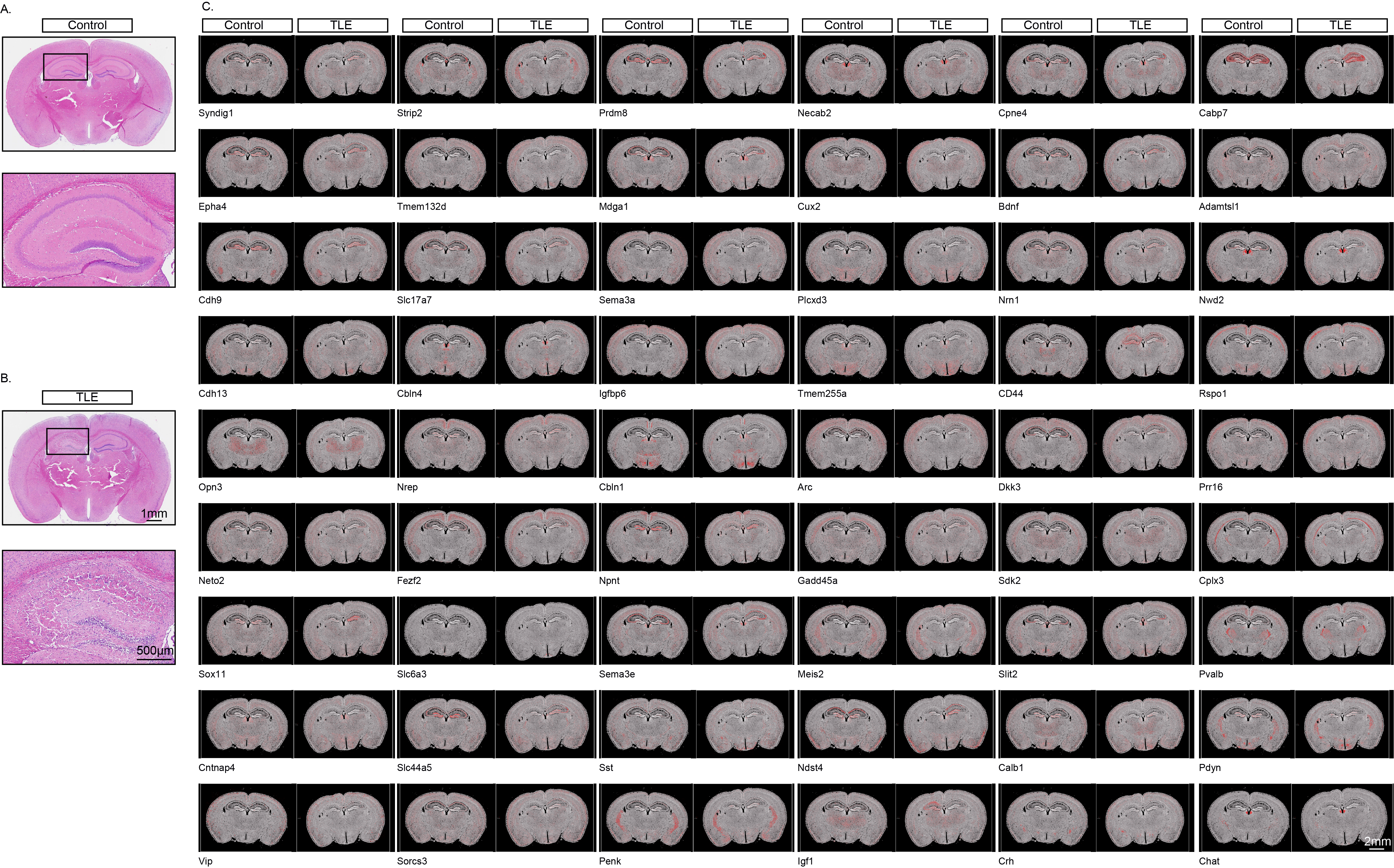

Supplement: Supplementary file 6 — Supplementary Material 6: Figure S6. A-B. Representative HE staining results of mouse brain sections from the Control group (A) and TLE group (B). The upper images display the whole brain at low magnification, while the lower images provide a high magnification view of the boxed region, specifically showing the ipsilateral hippocampus. C. High-resolution spatial expression distribution of the major marker genes used to define various neurons in control mouse brain sections. For each gene, the left side represents the Control group, while the right side represents the TLE group. [file 40364_2024_636_MOESM6_ESM.png]

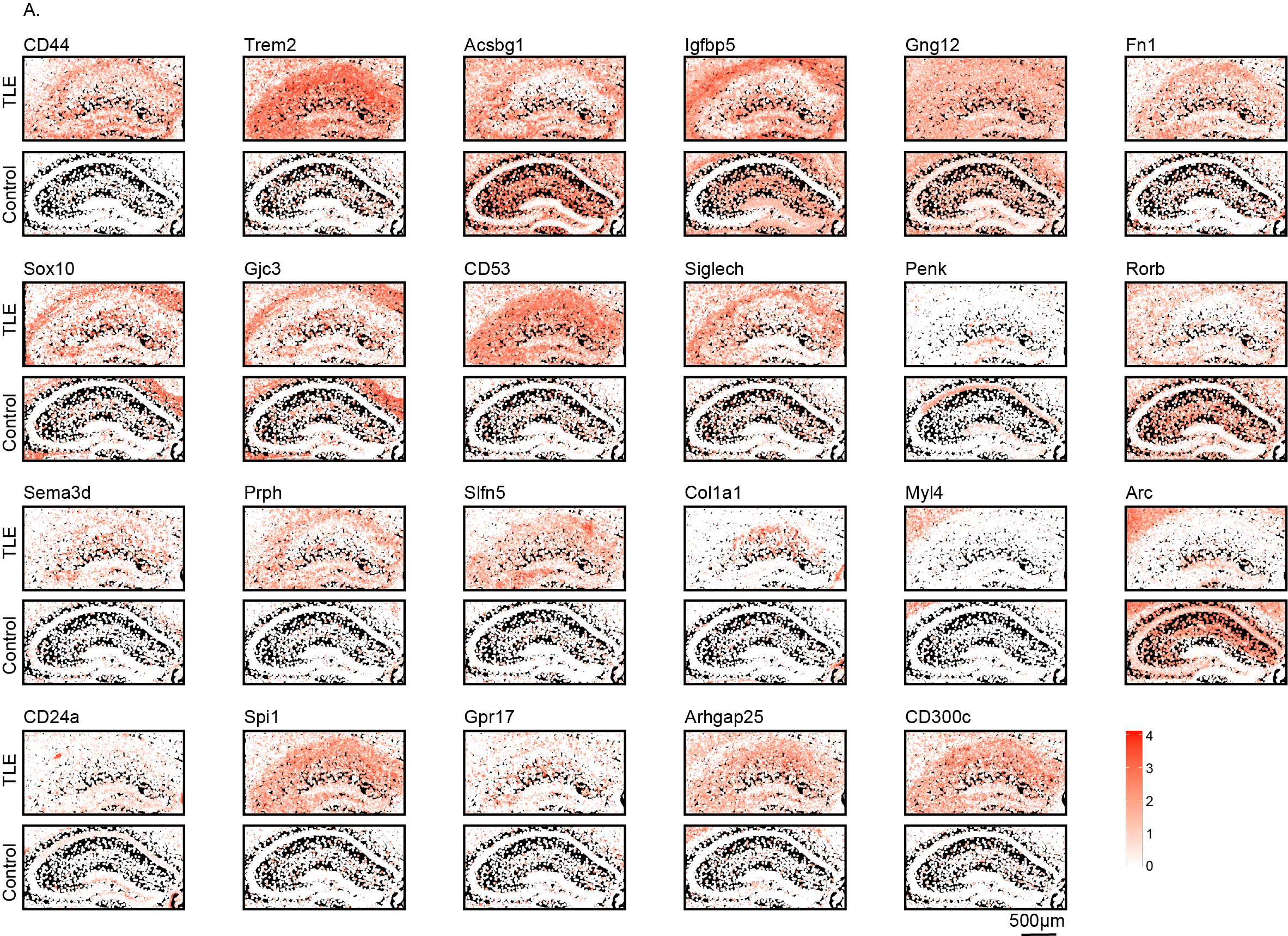

Supplement: Supplementary file 7 — Supplementary Material 7: Figure S7. A. Spatial expression distribution of the top 28 upregulated genes in ipsilateral hippocampus of TLE and control mouse. [file 40364_2024_636_MOESM7_ESM.png]

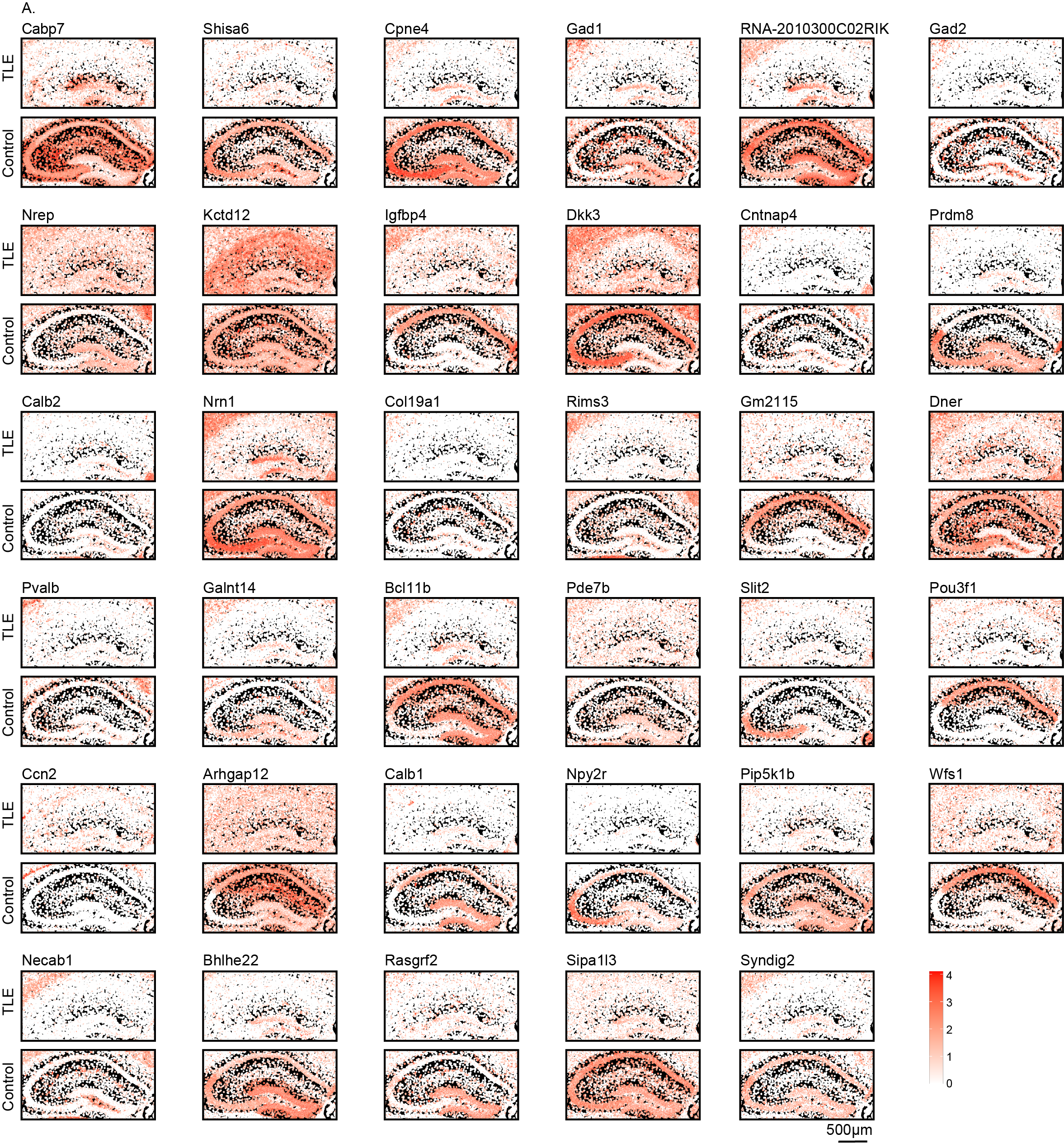

Supplement: Supplementary file 8 — Supplementary Material 8: Figure S8. A. Spatial expression distribution of the top 40 downregulated genes in ipsilateral hippocampus of TLE and control mouse. [file 40364_2024_636_MOESM8_ESM.png]
